# Diagnostic Potential of Exosomes in Colorectal Cancer: Current Advances and Future Perspectives

**DOI:** 10.3390/molecules31081339

**Published:** 2026-04-19

**Authors:** Kinga Suska, Marcin Piotrowski, Damian Jacenik, Jakub Fichna

**Affiliations:** 1 Department of Biochemistry, Faculty of Medicine, Medical University of Lodz, Mazowiecka 5, 92-215 Lodz, Poland; kinga.suska@umed.lodz.pl (K.S.); marcin.piotrowski1@student.umed.lodz.pl (M.P.); 2 Department of Cytobiochemistry, Faculty of Biology and Environmental Protection, University of Lodz, Pomorska 141/143, 90-236 Lodz, Poland; damian.jacenik@biol.uni.lodz.pl

**Keywords:** cancer diagnostics, colorectal cancer, exosome, exosomal biomarker, metabolomics, non-invasive biomarker

## Abstract

Colorectal cancer (CRC) remains one of the leading causes of cancer-related mortality worldwide and is frequently diagnosed at an advanced stage due to limitations of current screening methods. Although surgical resection is the standard treatment, conventional tissue biopsies are invasive and restrict real-time assessment of tumor dynamics. Liquid biopsy has emerged as a promising noninvasive approach enabling repeated analysis of tumor-derived components in body fluids. Among these, exosomes have gained considerable attention as potential diagnostic biomarkers in CRC. This review summarizes current evidence on exosome biogenesis, molecular composition, and their diagnostic relevance in colorectal cancer. We discuss exosomal nucleic acids, proteins, and lipids as biomarkers detectable in patient samples, as well as analytical platforms used for their isolation and characterization, including ultracentrifugation-based methods, size-exclusion chromatography, nanoparticle tracking analysis, electron microscopy, proteomics, lipidomics, and sequencing approaches. Accumulating data demonstrate that exosomal microRNAs, long non-coding RNAs, proteins, and lipid signatures correlate with tumor progression, immune modulation, angiogenesis, and epithelial–mesenchymal transition. Advances in microfluidic technologies, Raman/SERS spectroscopy, and AI-based data analysis are contributing to further improvements in diagnostic sensitivity and reproducibility. Despite their potential, the lack of standard isolation and validation protocols remains a major obstacle to clinical implementation, highlighting the need for large-scale multicenter studies before exosome biomarkers can be routinely used in CRC diagnostics.

## 1. Introduction

### 1.1. Colorectal Cancer

Colorectal cancer (CRC) is one of the most commonly diagnosed cancers worldwide, with more than 1.9 million cases in 2020 [1]. It ranks third in terms of the incidence of malignant tumors in Europe and America [2]. In developed countries, the incidence ranges from 30 to 70 cases per 100,000 people, while in regions with limited access to health care, the rate is 10 to 20 per 100,000 [3]. Despite a decline in the number of cases of CRC in the United States after the introduction of screening, there has been an increase in incidence among people aged 20–49, particularly in the 40–49 age group [4]. Similar trends have been observed in other developed countries [4]. The global burden of CRC is expected to increase up to 60% by 2030 [5]. In 2020, 930,000 people died from CRC, with the highest death rate in Eastern European men [1]. The pathogenesis of CRC is complex, involving genetic and epigenetic changes that lead to uncontrolled cell proliferation [6]. Most cases of sporadic CRC develop through an adenocarcinoma sequence, where a benign adenoma transforms into a malignant neoplasm [6]. The mechanisms include chromosomal instability (CIN), microsatellite instability (MSI) and the CpG island methylation phenotype (CIMP) [7].

**Figure 1:**
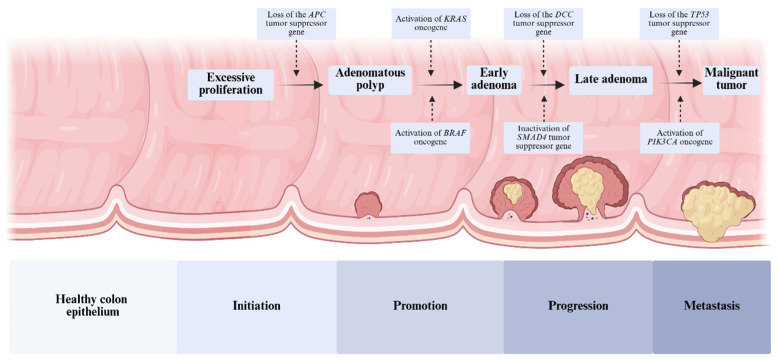
Development of colorectal cancer. Colorectal cancer develops in four stages: initiation, promotion, progression and metastasis. Most cases result from adenomatous polyps (APs), which are masses of dysplastic cells. When cancer cells penetrate the mucosa and invade the submucosa, the adenoma becomes malignant. Key mutations in the *APC*, *KRAS*, *BRAF*, *TP53* and *SMAD4* genes accelerate this process. Loss of APC function causes β-catenin accumulation, while *KRAS* and *BRAF* mutations activate the Ras-Raf-MEK-ERK pathway, promoting tumor growth. Subsequent mutations in *TP53*, *PIK3CA* and SMAD4 lead to invasion and metastasis, and loss of function of the *DCC* gene increases tumor aggressiveness [8,9,10,11,12,13].

Mutations in genes such as *APC*, *KRAS*, *BRAF*, *TP53* and *SMAD4* play a key role in the development of CRC [8]. Loss of APC function leads to β-catenin accumulation and activation of T cell factor (TCF)-dependent genes, which contributes to adenomas [8,9]. Mutations in the *KRAS* and *BRAF* genes activate the Ras-Raf-MEK-ERK signaling pathway, promoting cell growth and proliferation [10]. At later stages, mutations in *TP53*, *PIK3CA* and *SMAD4* cause the adenocarcinoma transition, increasing the risk of invasion and metastasis [11]. An important role in the process of tumor development is played by the *DCC* gene, whose loss causes uncontrolled cell proliferation and increases metastatic potential, promoting the migration and invasiveness of cancer cells [12,13,14] (Figure 1).

CRC is often detected only at an advanced stage, when treatment options are limited [3]. However, the implementation of primary prevention strategies, such as leading a healthy lifestyle, avoiding risk factors and regular screening, can significantly reduce the incidence and impact of this cancer [3].

### 1.2. Screening Methods for CRC

**Figure 2:**
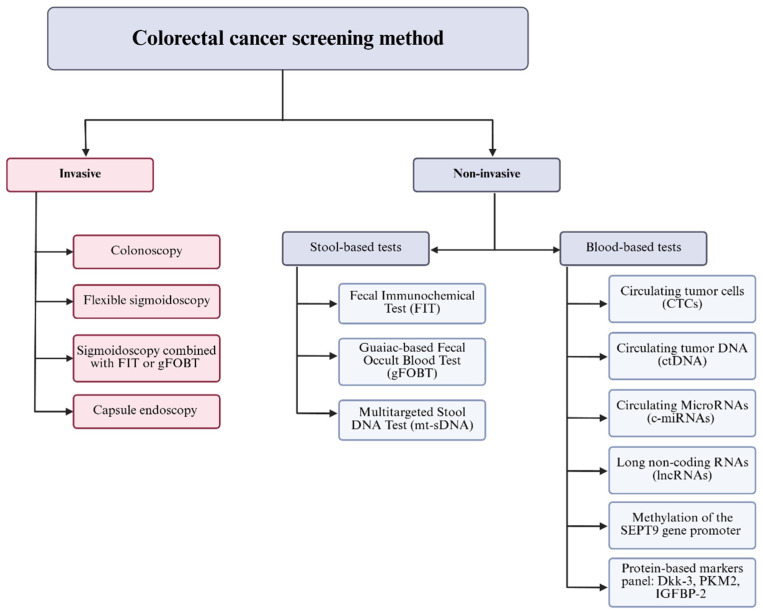
Colorectal cancer screening methods with clinically validated efficiency. Screening approaches are broadly classified into invasive and non-invasive techniques. Invasive methods include colonoscopy, flexible sigmoidoscopy, capsule endoscopy, and sigmoidoscopy combined with stool-based tests such as the fecal immunochemical test (FIT) or guaiac-based fecal occult blood test (gFOBT) [16,17,18]. Non-invasive strategies are further subdivided into stool-based tests—FIT, gFOBT, and the multitargeted stool DNA test (mt-sDNA)—and blood-based tests. Blood-based methods include detection of circulating tumor cells (CTCs), circulating tumor DNA (ctDNA), circulating microRNAs (c-miRNAs), long non-coding RNAs (lncRNAs), methylation of the *SEPT9* gene promoter, and panels of protein biomarkers including Dkk-3, PKM2, and IGFBP-2 [16,17,19,20,21,22,23,24,25,26].

There are several screening tests used for the detection of CRC and adenomatous polyps (AP) that vary in sensitivity, specificity, efficacy, convenience, safety, availability and cost [15] (Figure 2). Screening methods for CRC are divided into invasive and non-invasive approaches. Invasive methods include colonoscopy, flexible sigmoidoscopy, capsule endoscopy and sigmoidoscopy combined with the fecal immunochemical test (FIT) or guaiac-based fecal occult blood test (gFOBT) [16,17,18]. Non-invasive methods are classified into stool-based tests—such as FIT, gFOBT, and the multitargeted stool DNA test (mt-sDNA)—and blood-based tests, which include circulating tumor cells (CTCs), circulating tumor DNA (ctDNA), circulating microRNAs (c-miRNAs), long non-coding RNAs (lncRNAs), methylation of the *SEPT9* gene promoter, and protein-based biomarker panels (including Dickkopf-3 (Dkk-3), Pyruvate kinase M2 (PKM2), and Insulin-like growth factor binding protein 2 (IGFBP-2)) [16,17,19,20,21,22,23,24,25,26].

Recent advances also explore multi-omics approaches that combine genomic, epigenetic, and proteomic biomarkers to improve diagnostic accuracy [27,28,29]. Furthermore, artificial intelligence (AI) and machine learning algorithms are being integrated into image analysis and risk prediction models, aiming to personalize CRC screening in the near future [16,30,31,32].

#### 1.2.1. Invasive Colorectal Cancer Screening

Colonoscopy remains the gold standard for CRC screening due to its high sensitivity and specificity. It allows for the visualization and removal of neoplastic and pre-cancerous lesions throughout the entire colon, serving as both a diagnostic and therapeutic tool, particularly when other screening tests yield positive results [18,33,34,35,36]. While considered relatively safe, with a perforation rate below 1 per 1000 cases—typically due to polypectomy—it requires full bowel preparation and sedation, which can limit patient compliance [18,33,34,35,36].

Despite its widespread availability, colonoscopy is resource-intensive and not easily scalable for mass screening due to its cost and the need for trained personnel [37,38,39,40]. Multiple observational studies and meta-analyses have shown substantial reductions in CRC mortality among those who undergo colonoscopy—ranging from 68% to 88%—compared to those who do not [18,40,41,42]. However, this benefit is not uniform throughout the colon. Studies report significantly reduced mortality from distal CRC (up to 47%), but minimal or no benefit in the proximal colon [18,38]. This may be attributed to factors such as incomplete procedures, variations in endoscopist skills, inadequate bowel preparation, and anatomical or biological differences in tumor behavior between proximal and distal sites [43,44,45].

In contrast, flexible sigmoidoscopy (FS) requires less bowel preparation and can be performed without sedation. Several randomized controlled trials have demonstrated that FS, with follow-up colonoscopy if lesions are detected, significantly reduces CRC mortality [46,47]. Pooled analyses have shown a 26–31% reduction in overall CRC mortality, and up to 46% for tumors located in the distal colon [19,20,43,44,45]. Nevertheless, its limited reach (restricted to the rectum and sigmoid colon) has led many screening programs to favor colonoscopy for its comprehensive protection.

#### 1.2.2. Non-Invasive Colorectal Cancer Screening

##### Stool-Based Tests

gFOBTs detect fecal hemoglobin through heme pseudoperoxidase activity based on the color change of paper soaked in guaiac reagent [48]. However, peroxidase found in plant foods, heme found in meat foods, as well as non-steroidal anti-inflammatory drugs (NSAIDs) and anticoagulants can cause false-positive gFOBT results. On the other hand, consuming large amounts of vitamin C can cause a false negative gFOBT result [48,49]. Despite these limitations, gFOBTs have been widely used for decades due to their low cost and ease of use [48,49,50]. The sensitivity of the test for CRC ranges from 51% to 100% and specificity from 90% to 97% [51], and Hewitson’s screening study showed a reduction in the relative risk of CRC mortality of 16% [52] and up to 25% in the Minnesota Colon Cancer Control Study [53]. However, the need for dietary restrictions and multiple stool samples, as well as poor sensitivity and low specificity, have led to their replacement by FITs in many screening programs [20]. FITs are now preferred for CRC screening due to their higher accuracy, automation and adjustable test cutoff thresholds [19,21]. World Health Organization (WHO) and European guidelines recommend FITs, although gFOBTs are still used in some combined strategies [54].

FITs are highly specific methods for detecting human fecal hemoglobin using specific antibodies, with a United States Food and Drug Administration (FDA)-approved positivity threshold of 20 μg/g [48]. FITs offer several advantages, including higher specificity (96.4%) compared to multi-target stool DNA (mt-sDNA) tests (89.8%) [21] and lower cost [55]. Designed for ease of use, FITs are shipped directly to patients for home sampling, making them suitable for mass screening programs and compliant with all screening guidelines [56]. On the other hand, sensitivity for FIT can reach 97.0% at the lowest cutoff of 2 µg/g, making it possible to rule out CRC with a negative test result, while a positive result is more effective than relying on symptoms alone to select patients for further diagnosis [57]. FITs have also the advantages of affordability, accessibility and the ability to manipulate the cutoff point for test sensitivity [19].

The mt-sDNA test combines FIT for hemoglobin with detection of methylated DNA and mutations associated with CRC [58]. This approach aims to increase the sensitivity of detecting CRC and advanced adenomas [58]. In a study of 9989 people undergoing colonoscopy, the mt-sDNA test showed 92% sensitivity for CRC and 42% for advanced adenomas, outperforming FIT (74% sensitivity for CRC, 24% for advanced adenomas) [59]. However, mt-sDNA showed lower specificity (87%) compared to FIT (95%) [21]. Despite its higher sensitivity, mt-sDNA faces several limitations. Its high cost makes it less cost-effective than FIT or colonoscopy [60,61]. Additionally, the lower specificity of mt-sDNA increases the likelihood of false positives, raising concerns about over-testing and unnecessary follow-up testing after normal colonoscopy results [62,63]. Although mt-sDNA increases detection capabilities, its high cost, complexity and specificity limit its practicality compared to simpler and more cost-effective methods such as FIT or gFOBT [64].

##### Blood and Liquid Biopsy-Based Tests

Blood tests are becoming promising tools for early detection and monitoring of CRC [26,65,66]. Unlike invasive procedures such as colonoscopy or biopsy [67,68,69], blood tests are considered non-invasive, meaning they do not require penetration of internal body cavities or tissues [26,65]. This results in a lower risk of complications, such as perforation, bleeding or infection, and does not require anesthesia or bowel preparation, making it more acceptable and accessible to patients [70,71,72]. The relative ease of blood collection, combined with advances in molecular diagnostic techniques, allows the detection of a variety of circulating biomarkers, including proteins, tumor DNA, tumor-derived cells and non-coding RNA [66,73]. In addition, analytical techniques used in blood tests—such as polymerase chain reaction (PCR) [74,75,76], next-generation sequencing (NGS) [77,78] and Raman spectroscopy [79] allow accurate analysis of tumor biomarkers with high sensitivity and specificity. Despite their many advantages, blood tests still need standardization, validation and more population-based testing before they can become a routine part of screening programs [65,80,81,82]. However, their development represents a promising step toward a more accessible, convenient and safe diagnosis of CRC. As a result, there is a growing interest in blood biomarkers that offer a less invasive and potentially more acceptable approach to CRC screening [65].

Liquid biopsy enables the analysis of tumor-derived materials, such as CTCs, and cell-free DNA (cfDNA), mostly from peripheral blood [22,83]. This minimally invasive approach is widely accepted by patients and has shown potential in early CRC detection, minimal residual disease monitoring, and therapeutic guidance [84,85,86]. Despite these advantages, current limitations include the need for greater standardization and validation before broad clinical implementation [87].

CTCs originate from primary or metastatic CRC lesions and can be found in peripheral blood, offering insight into tumor dissemination and therapeutic response [84]. However, the rarity of CTCs in blood (often 1–10 cells per 10 mL) limits diagnostic reliability [88]. Newer technologies, such as microfluidic devices and size-based isolation methods, aim to improve sensitivity [89]. While the prognostic value of CTCs is supported, their utility in screening as of today remains controversial [84,90].

ctDNA is a subset of cfDNA released by tumor cells through apoptosis or necrosis and harbors cancer-specific genetic and epigenetic alterations [91]. These fragments reflect mutations, methylation changes, and microsatellite instability, making ctDNA a powerful biomarker [91,92]. Sensitivity and specificity of ctDNA for CRC diagnosis are high, but despite promising diagnostic potential, further standardization is required [80,93].

C-miRNAs are stable, non-coding RNA molecules involved in post-transcriptional gene regulation [94,95,96]. Although many miRNAs are dysregulated in CRC [97,98], their use as single biomarkers faces challenges due to low specificity and sensitivity [99]. Technologies such as quantitative reverse transcription polymerase chain reaction (RT-qPCR) and NGS allow profiling of miRNA panels, but lack of standardized protocols, normalizers, and control for hemolysis remain barriers to clinical adoption [100,101,102].

Another promising group of biomarkers includes lncRNAs, such as colon cancer-associated transcript 1 (*CCAT1)* and HOX transcript antisense RNA (*HOTAIR)*, which are upregulated in CRC patients [103,104]. These molecules are involved in the regulation of key signaling pathways (e.g., WNT/β-catenin) [23,105]. Despite their potential, clinical application is currently hindered by challenges in extraction, lack of standardization, and insufficient normalization strategies [106].

Among protein-based biomarkers, insulin-like growth factor binding protein 2 (IGFBP-2) is overexpressed in CRC and correlates with tumor progression and serum carcinoembryonic antigen (CEA) levels [25,107,108]. Similarly, the glycolytic enzyme pyruvate kinase M2 (PKM2) is upregulated in CRC and detectable in both blood and stool; however, its low specificity limits its utility as a standalone marker [109,110,111]. Dkk-3, a member of the Dickkopf glycoprotein family, plays a pro-angiogenic role in CRC and is associated with tumor vascularization [112,113]. Its epigenetic silencing has been implicated in CRC progression, but further validation is needed to confirm its diagnostic relevance [113,114].

Notably, a panel combining Dkk-3, PKM2, and IGFBP-2 has shown a sensitivity of 57% for stage I and 76% for stage II CRC at 95% specificity, suggesting its potential as a non-invasive alternative to current screening methods such as FOBT and FIT [115].

## 2. Exosomes

### 2.1. Structure and Characteristics of Exosomes

Extracellular vesicles (EVs) are formed by active secretion, and their formation can result from direct detachment from the cell membrane or by fusion of larger multi-vesicular structures [5]. Among EVs we can find, among others, exosomes (30–150 nm), microvesicles (150–1000 nm), apoptotic vesicles (100–1000 nm), apoptotic bodies (1–5 μm), and large oncosomes (1–10 μm) [116].

Regarding the maximum size of exosomes, scientific sources usually agree by giving a value of 150 nm [116,117,118,119,120], but the minimum size is determined in the range of 30–50 nm [116,117,118,119,120,121]. The techniques used to measure the size of exosomes and the storage conditions of the samples may be mainly responsible for the differences [122]. Morphological heterogeneity and different origins of cells producing exosomes do not seem to have a significant impact on their size [122]; however, these aspects are crucial in defining their function, content and other characteristics and properties [123,124].

**Figure 3:**
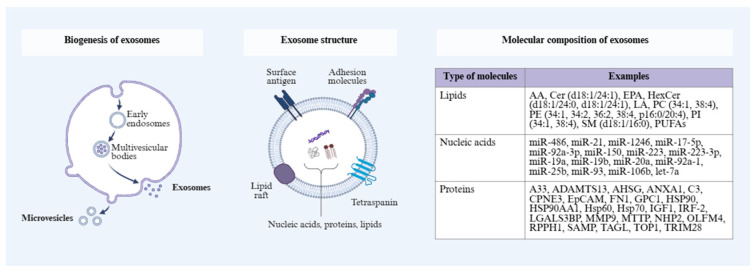
Biogenesis and molecular composition of exosomes. Exosomes are formed via the endosomal pathway, which begins with endocytosis and the formation of early endosomes that mature into multivesicular bodies (MVBs). As a result of MVB fusion with the cell membrane, intercellular vesicles are released in the form of exosomes, while alternative pathways lead to the formation of microvesicles through direct membrane budding. Structurally, exosomes are surrounded by a lipid bilayer enriched with surface antigens, adhesion molecules, and tetraspanins, and lipid rafts contribute to membrane organization. Exosomes carry a diverse cargo of biomolecules, including lipids, nucleic acids and proteins, which reflect the physiological and pathological state of cells and participate in intercellular communication [125,126,127,128]. Abbreviations: AA, arachidonic acid; ADAMTS13, ADAM metallopeptidase with thrombospondin type 1 motif 13; AHSG, alpha-2-HS glycoprotein; ANXA1, annexin A1; C3, complement component 3; Cer, ceramide; CPNE3, copine III; EpCAM, epithelial cell adhesion molecule; FN1, fibronectin 1; GPC1, glypican-1; HSP90, heat shock protein 90; HSP90AA1, heat shock protein 90 alpha family class A member 1; Hsp60, heat shock protein 60; Hsp70, heat shock protein 70; IGF1, insulin-like growth factor 1; IRF-2, interferon regulatory factor 2; LA, linoleic acid; LGALS3BP, lectin galactoside-binding soluble 3 binding protein; MMP9, matrix metalloproteinase 9; MTTP, microsomal triglyceride transfer protein; NHP2, H/ACA ribonucleoprotein complex subunit 2; OLFM4, olfactomedin 4; PC, phosphatidylcholine; PE, phosphatidylethanolamine; PI, phosphatidylinositol; PUFAs, polyunsaturated fatty acids; RPPH1, ribonuclease P RNA component H1; SAMP, small archaeal modifier protein; SM, sphingomyelin; TAGL, transgelin; TOP1, DNA topoisomerase 1; TRIM28, tripartite motif containing 28.

Endocytosis is the beginning of exosome biogenesis, with an inward budding of the plasma membrane and formation of early endosomes. The maturation of these structures results in the formation of multivesicular bodies (MVBs). Endosomal sorting complex required for transport (ESCRT) is responsible for the formation of intraluminal vesicles (ILVs) inside MVBs—it is in MVBs that the sorting of biomolecules takes place. The fate of maturing MVBs can end in various ways. Among the most important possibilities, we can include the fusion of MVBs with lysosomes and their degradation, and their fusion with the plasma membrane, resulting in the release of ILVs as exosomes into the extracellular space [125,126,127,128] (Figure 3). Exosomes are secreted by most cell types [125,129]—including cancer cells [123]—and can be isolated from body fluids such as blood, urine, cerebrospinal fluid, synovial fluid, breast milk or saliva, among others [125].

Within the tumor microenvironment (TME), exosomes are key mediators of cell-to-cell communication, facilitating interactions between the cancer cells and surrounding stromal elements, including cancer-associated fibroblasts (CAFs) and immune cells. These vesicles deliver biologically active cargo, such as miRNAs and proteins, which can alter the behavior of recipient cells and drive tumor development [130]. For example, in colorectal cancer (CRC) exosomal miR-21 has been reported to stimulate CAF activation, increasing the release of pro-tumorigenic factors like hepatocyte growth factor (HGF) and stromal-derived factor 1 (SDF-1), thereby promoting extracellular matrix remodeling and invasion [131]. At the same time, CRC-derived exosomes influence immune responses by inducing macrophage polarization toward the M2 phenotype via mediators such as miR-155 and TGF-β, contributing to an immunosuppressive environment. In addition, exosomes carrying pro-angiogenic factors, including vascular endothelial growth factor (VEGF), can activate endothelial cells and enhance angiogenesis [132,133]. These observations highlight that exosomes function not only as diagnostic biomarkers but also as active contributors to tumor progression, metastasis, and therapeutic resistance.

### 2.2. Exosome Isolation and Characterization

Various methods have been developed for the isolation of exosomes, each with its own strengths and weaknesses [134]. The most commonly used technique is differential ultracentrifugation, which involves stepwise centrifugation to separate exosomes based on their size and density [134]. Density gradient centrifugation provides higher purity, but typically results in lower yields than ultracentrifugation [134]. Size exclusion chromatography separates vesicles by size, which provides good purity, but often at the expense of efficiency [134]. Immunoaffinity capture relies on antibodies that bind to the surface proteins of exosomes, allowing specific isolation [134]. Polymer-based precipitation is a simple method, but can lead to co-isolation of impurities [134].

Characterizing exosomes requires several key techniques, such as electron microscopy, nanoparticle tracking analysis (NTA), dynamic light scattering (DLS), flow cytometry, Western blot, RNA sequencing, atomic force microscopy (AFM) and Raman spectroscopy [134]. Among these methods, NTA is one of the most widely used and cited [134]. Together, these techniques provide a comprehensive understanding of the properties of exosomes, and the choice of method depends on the purpose of the study [134].

### 2.3. Exosomes as Potential Biomarkers in Oncological Diagnosis

Exosomes are distinguished by the wealth of biomolecules they contain. The content of exosomes reflects the environment of the cells from which they originate, so they can provide information on the physiological or pathophysiological state of these cells [123,135,136,137]. They harbor proteins, lipids and nucleic acids, such as RNA, which can serve as specific biomarkers for various types of cancer [138,139]. The diagnostic and therapeutic potential of exosomes also stems from their ability to regulate physiological and pathological processes, such as the immune response, cell signaling and cancer development [6]. By transferring and exchanging metabolites and signaling molecules between cells, exosomes can influence key steps in carcinogenesis, including cell proliferation, changes in the tumor microenvironment (TME), metastasis and invasion [5]. Studies show that exosomes can promote tumor growth [7,140], evade the immune response [141], promote angiogenesis [142,143], facilitate metastasis [142,144,145], contribute to chemotherapy resistance [146] and induce endothelial–mesenchymal transition (EMT) in target cells [147,148,149].

Taken together, exosomes can enable early identification of tumors and provide information on tumor characteristics and behavior, which is important in developing appropriate therapeutic strategies [138,139]. For this reason, exosomes have gained considerable attention in recent years as potential biomarkers in cancer diagnosis [147]. Their growing importance stems from—among others—their ability to provide valuable diagnostic information in a non-invasive manner, making them a promising tool in medicine [140]. They can be isolated from body fluids, allowing safe and easy collection of diagnostic material [150,151]. This feature is particularly important for patients who may poorly tolerate invasive procedures such as biopsies. With the ability to monitor changes in the patient’s body on a regular basis, exosomes can significantly facilitate tracking disease progression and assessing response to treatment [150,151].

Another advantage of exosomes is their ability to be monitored in real time. Because exosomes can reflect dynamic changes in the TME, their analysis can be used to track tumor progression and response to treatment on an ongoing basis [139,151]. This ability to track disease in real time supports the development of personalized medicine, where treatment can be tailored to a patient’s individual tumor profile [139,151]. Moreover, studies indicate that some exosomal biomarkers can be detected as early as the earliest stages of cancer, which can significantly improve early detection rates and thus increase patients’ chances of successful treatment [150,151].

### 2.4. Exosomal Biomarkers

#### 2.4.1. Lipids

Abnormal lipid metabolism is recognized as a hallmark of cancer cells, contributing to both tumor-promoting and tumor-suppressing effects depending on the cellular context [152]. Lipids not only maintain the structural integrity of cancer cell membranes but also actively participate in cellular signaling pathways and oncogenic processes [153]. Importantly, they serve as key components of exosomes, that facilitate intercellular communication and play crucial roles in the TME [153,154].

The lipid bilayer of exosomes is particularly enriched with phosphatidylserine (PS), sphingomyelin (SM), phosphatidic acid (PA), ceramides (Cer), and cholesterol [155]. These lipids are critical not only for the structural stability of exosomal membranes but also for the processes of exosome biogenesis, cargo loading, and release from parent cells [155]. Furthermore, exosomal lipids contribute to dynamic cellular interactions by forming ‘mobile rafts’—specialized lipid domains that can transform exosomes into extracellular “signosomes,” thereby spreading signaling pathways essential for carcinogenesis and metastasis [153]. Ceramides, in particular, regulate the assembly and function of these mobile rafts, influencing key intracellular signaling cascades [153].

Beyond their structural roles, exosomal lipids such as Cer and phosphatidic acid act as second messengers, initiating signaling pathways involved in metabolic reprogramming, tumor progression, and the development of drug resistance [156,157]. For example, synthetic lipid-enriched nanoparticles designed to mimic the composition of exosomal membranes have been shown to activate the NF-κB/SDF-1α signaling axis, thereby enhancing survival pathways in pancreatic cancer cells [158,159,160]. Moreover, lysophosphatidic acid (LPA), produced by autotaxin (ATX) within exosomes, has been implicated in cancer-associated pain and the promotion of tumor progression through activation of LPA receptors on sensory neurons [161].

Exosomal lipids also influence ferroptosis, an iron-dependent form of cell death characterized by lipid peroxidation [162]. Exosomes derived from adipose tissue have been shown to inhibit ferroptosis in CRC cells, contributing to chemotherapy resistance by upregulation of ferroptosis inhibitors such as microsomal triglyceride transfer protein (MTTP) [162].

Exosomal lipids also play a crucial role in modulating immune responses within the TME. Sphingosine-1-phosphate (S1P) and PS, among others, have been shown to suppress T cell receptor signaling and impair dendritic cell function, promoting immune evasion and supporting tumor progression [148,163,164,165]. In addition, glycolipids and prostaglandins—particularly prostaglandin E2 (PGE2)—carried by tumor-derived exosomes further facilitate immunosuppression and metastatic niche formation [166,167].

Currently, there is growing interest in the use of exosomal lipids as potential cancer biomarkers [168]. However, lipidomic studies have been conducted on exosomes derived from ovarian, breast, and prostate cancer cell lines [163,168,169], and the research focusing specifically on CRC exosomal lipids remains limited.

##### Exosomal Lipid Signatures in CRC

Bestard-Escalas et al. [170] analyzed changes in the exosomal lipid composition among individuals with various colorectal lesions—hyperplastic polyps (HP), AP, invasive neoplasia (Neo), and hereditary non-polyposis colorectal cancer (Her)—compared to healthy controls. Their study revealed that phosphatidylcholine (PC) levels were consistently elevated in diseased groups, achieving statistical significance specifically in patients with Her and AP. On the other hand, SM showed a decreasing trend, with significant reduction noted only in the Her group. Detailed lipid profiling highlighted a pattern: species containing a single monounsaturated fatty acid (MUFA) such as 34:1, 36:1, or 38:1 (where the first number indicates the carbon atoms and the second the number of double bonds) were reduced, while lipids enriched with di- or polyunsaturated fatty acids, including 34:2, 36:4, 36:3, 36:2, and 38:4, were elevated in pathological samples. Consistently across all disease groups, levels of PC 34:1, phosphatidylethanolamine (PE) 34:1, and phosphatidylinositol (PI) 34:1 were decreased, whereas PC 38:4, PE 38:4, and PI 38:4 were increased compared to healthy controls. Based on this lipid remodeling, the researchers proposed the 34:1/38:4 ratio as a novel diagnostic biomarker for CRC, demonstrating a sensitivity of 54.6%, specificity of 94.4%, and a positive predictive value of 96%.

##### Stage-Dependent Variations in Exosomal Profiles

Expanding on the connection between lipid profiles and disease progression, Elmallah et al. [171] examined whether lipid alterations in exosomes correlate with metastatic status of CRC. Their findings showed that levels of sphingomyelin (SP) and PC were increased in all analyzed exosomes. In patients without metastases and in non-metastatic cells (HCT116), significant increases were observed in lipids such as PC 34:1, PE 36:2, SM d18:1/16:0, and hexosylceramide (HexCer) d18:1/24:0 and d18:1/24:1, when compared to healthy controls and normal colonic mucosa cells (NCM460D).

In contrast, exosomes derived from metastatic CRC and SW620 cells presented reduced levels of phosphorylated PE 34:2, PE 36:2, and phosphorylated PE p16:0/20:4. Meanwhile, the Cer d18:1/24:1 was elevated.

These findings suggest that PE 34:2, PE 36:2, phosphorylated PE p16:0/20:4, and Cer d18:1/24:1 may serve as potential indicators of metastasis in CRC, whereas PC 34:1, PE 36:2, SM d18:1/16:0, and HexCer species could differentiate primary tumor patients from healthy individuals. However, further validation in larger clinical cohorts is necessary to establish their diagnostic utility.

##### Exosome-Associated Inflammatory Biomarkers in CRC Development

Lipidomic analysis revealed a significantly higher accumulation of polyunsaturated fatty acids (PUFAs) in human colon adenocarcinoma cells (Caco-2) relative to non-cancerous colon epithelial cells (HCEC-1CT), with omega-6 fatty acids (FA) being the most prevalent [172]. In contrast, the exosomes derived from Caco-2 cells contained lower levels of omega-3 FAs—specifically α-linolenic acid (ALA) and eicosapentaenoic acid (EPA)—when compared to those from HCEC-1CT cells. Furthermore, exosomes from the Caco-2 cells exhibited an enrichment in pro-inflammatory lipids such as γ-linolenic acid (GLA), linoleic acid (LA), and arachidonic acid (AA). Notably, the elevated n-6/n-3 FAs and AA/EPA ratios observed in Caco-2 cells exosomes suggest a shift toward a pro-inflammatory lipid profile, supporting the hypothesis that such imbalances may contribute to CRC development.

#### 2.4.2. miRNA

Although the proportion of miRNAs in exosomes can vary widely depending on physiological condition, tissue type or cell type, they are often among the most common RNA molecules present in exosomes [173,174]. Exosomes have a protective function for miRNAs, ensuring their stable presence in the extracellular space and integration by specific recipient cells [175]. It has been shown that miRNAs differentially expressed in exosomes found in blood of CRC patients may be potential biomarkers for cancer diagnosis [176].

##### Exosomal miRNA Signatures in CRC

Liu et al. [177] documented increased levels of miR-486 in plasma exosomes of CRC patients, which may indicate its use as a potential biomarker for CRC diagnosis [177]. Interestingly, the suppressive effect of miR-486-5p through activation of the PLAGL2/IGF2/β-catenin signaling pathway has been pointed out, which may become a therapeutic target for CRC treatment.

Another study revealed that high levels of a series of miRNAs—miR-17, -18a, -19a, -19b-1, -20a and -92a-1—and miRs—miR-25b, -93 and -106b—were detected in exosomes isolated from the LIM1863 CRC cell line culture [178]. In contrast, higher levels of miR-19a, -19b and -92a expression were found in the exosomes of CRC patients, which the authors linked to the recurrence of liver metastases [179].

##### Stage-Dependent Variations in Exosomal Profiles

Earlier studies have shown that the levels of seven miRNAs found in serum exosomes (let-7a, miR-1229, miR-1246, miR-150, miR-21, miR-223 and miR-23a) were significantly higher with primary CRC, as well as with early-stage CRC, compared to control group [180]. In contrast, the levels of these miRNAs were significantly reduced after surgical resection of the tumor. In addition, colon cancer cell lines showed significantly higher levels of these miRNAs compared to cell lines derived from normal colon epithelium.

These observations were later partially confirmed, by observing elevated levels of let-7a in the serum of CRC patients compared to healthy patients [181]. Moreover, let-7a was found to affect synaptosomal-associated protein 23 (SNAP23)-mediated inhibition of EVs and mitochondrial oxidative phosphorylation (OXPHOS) secretion, thereby affecting the inhibition of CRC progression. To note, OXPHOS provides essential ATP for CRC cell growth and supports tumor initiation and progression [182,183]. By switching between glycolysis and OXPHOS in response to stress, CRC cells maintain metabolic flexibility that promotes survival, invasion, and therapy resistance [184,185,186]. With this observation, it can be considered that the let-7a/SNAP23 axis may become a source of cancer biomarkers and represent new therapeutic targets.

Another study revealed elevated levels of exosomal miR-17-5p and miR-92a-3p, which were associated with CRC stages and grade [187]. Thus, they could be considered promising biomarkers of primary and metastatic CRC.

##### Exosome-Associated Inflammatory Biomarkers in CRC Development

Cooks et al. [188] demonstrated higher levels of miR-1246 in the exosomes obtained from CRC patients with *TP53* mutation (mutp53) compared to cancer patients without mutp53. With this information, they concluded that miR-1246 is associated with the occurrence of *TP53* mutations in CRC cells. In addition, CRC cells with mutp53 gain of oncogenic functions (GOF) were found to release exosomes containing miR-1246, which contributes to macrophage reprogramming. This indicates that miR-1246 is involved in inflammation and modulation of the TME.

miR-223-3p is another miRNA that may become a biomarker of inflammation in CRC. Bao et al. [189] showed that the overexpression of exosomal miR-223-3p promotes the M2 phenotype of macrophages, leading to IL-17 secretion, resulting in increased proliferation and migration of CRC cells.

#### 2.4.3. Proteins

There is now a growing interest in proteomic technology, through which the composition and functions of proteins that are contained in exosomes have been studied [185]. Recent reports demonstrated that exosomal proteins exhibit different levels of expression in different types of cancer, making them useful in the prediction, diagnosis and progression of cancer [186]. Proteins such as carbohydrate antigen 19-9 (CA19-9) and CEA are currently used to distinguish cancers of the liver, pancreas or stomach, but they cannot be used to diagnose CRC due to their low sensitivity, especially at an early stage [190].

##### Exosomal Proteins Signatures in CRC

Palmqvist et al. [191] identified 36 proteins whose expression levels were elevated in exosomes purified from serum of CRC patients compared to healthy subjects, including fibronectin 1 (FN1), annexin A1 (ANXA1), galectin-binding protein-3 (LGALS3BP), matrix metalloproteinase-9 (MMP9), A disintegrin, and metalloproteinase with thrombospondin motifs 13 (ADAMTS13) proteins, and 22 proteins whose expression levels were downregulated, including insulin-like growth factor I (IGF1), human stress-inducible 90 kDa heat shock protein alpha (HSP90AA1), complement component 3 (C3), and α2-HS Glycoprotein (AHSG). It was noted that FN1 controls the activity of 18 proteins responsible for cytoskeletal structure and integrin signaling during tumor development and metastasis, while IGF1, HSP90AA1 and C3 interact with proteins involved in maintaining tumor cell adhesion. Concurrently, Campanella et al. [192] revealed significantly higher levels of heat shock protein 60 (Hsp60) in the membrane of exosomes of CRC patients before tumor resection, while no such proteins were found in exosomes of patients after surgery, indicating that Hsp60 may be a promising biomarker for detecting the presence of colon adenocarcinoma [192]. Another study suggested classifying glipican-1 (GPC1) found in exosomes as a biomarker to diagnose patients with CRC [193]. The percentage of exosomes showing the presence of GPC1 and the expression level of GPC1 protein in the exosomes of patients before surgery were significantly increased, and after surgery both indicators returned to typical values. In contrast, Liang et al. [194] documented higher levels of exosomal ribonuclease P RNA component H1 (RPPH1) protein in the plasma of CRC patients without surgery, while the levels of this protein decreased after tumor removal. Another diagnostic and prognostic biomarker turned out to be copine protein III (CPNE3), whose levels were elevated in the plasma exosomes of CRC patients [195]. Interestingly, it has been shown that by combining two biomarkers—CPNE3 and CEA—a better ability to detect CRC can be obtained. In another study, bioinformatics analysis identified six exosomal proteins—NHP2 ribonucleoprotein (NHP2), olfactomedin 4 (OLFM4), topoisomerase 1 (TOP1), small archaeal modifier protein (SAMP), transgelin (TAGL) and tripartite motif-containing 28 (TRIM28), that differed in expression between CRC and the neighboring tissues [196], indicating that all of these proteins could be potential biomarkers to distinguish CRC tissues from healthy tissues [196]. Finally, Zhang et al. demonstrated increased expression of MTTP derived from plasma exosomes of CRC patients with high adipose tissue [192]. This study also documented that MTTP reduces the susceptibility of cells to ferroptosis, resulting in reduced sensitivity to chemotherapy. It has been suggested that targeting treatment to secreted MTTP may help combat oxaliplatin resistance in CRC [162].

##### Stage-Dependent Variations in Exosomal Profiles

Rai et al. [197] emphasized the importance of exosomes in CRC progression, where they are responsible for fibroblast activation, which results in increased expression of proteins that determine cell growth, invasion and metastasis.

Sun et al. [198] showed that high levels of interferon regulatory factor-2 (IRF-2) expression in serum exosomes were seen in CRC patients who had lymph node metastasis, in contrast to those without metastasis and healthy controls. IRF-2 is involved in remodeling of the lymphatic network of lymph nodes, thereby facilitating metastasis.

An association between exosomal RPPH1 overexpression and advanced stages of TNM Classification of Malignant Tumors (stages III and IV), and worse prognosis has been demonstrated [190]. RPPH1 through interaction with tubulin β-III (TUBB3) prevents its ubiquitination, thus RPPH1 may be involved in EMT of CRC cells [199]. With the information cited, it is possible to classify RPPH1 not only as a potential diagnostic biomarker, but also as a therapeutic target [199].

##### Exosome-Associated Inflammatory Biomarkers in CRC Development

Exosomal proteins play a crucial role in modulating inflammation in CRC by mediating communication between tumor cells and the TME [130]. Exosomes released by CRC cells contain both inhibitory and stimulatory molecules that alter the immune system balance, favoring the expansion and recruitment of regulatory T cells (Tregs) and myeloid-derived suppressor cells (MDSCs), or by blocking the activity of CD8^+^ T cells, dendritic cells (DCs), and natural killer (NK) cells [130].

Specific exosomal proteins such as epithelial cell adhesion molecule (EpCAM) [200,201] and transmembrane glycoprotein A33 (A33) [200,202,203] are highly expressed in CRC-derived exosomes and are involved in immunomodulation and cell adhesion, further influencing the TME. In addition, Hafez et al. [204] showed that EpCAM is involved in the regulation of EMT, a process that enhances cancer cell invasiveness and is associated with inflammatory signaling in the TME [204]. In fact, EpCAM-containing exosomes can modulate the immune response by influencing the phenotype and function of immune cells, thereby contributing to immune evasion and chronic inflammation in CRC [200,201,204].

Mathivanan et al. conducted a proteomic analysis of A33-positive exosomes from CRC cells which revealed a tissue-specific protein signature, including proteins involved in signaling, trafficking, and cytoskeletal organization, many of which are implicated in inflammation and tumor growth [205]. Notably, A33-exosomes are enriched in molecules associated with antigen presentation, such as MHC class I proteins, suggesting a potential role in modulating immune surveillance and inflammatory responses in the tumor microenvironment [200]. The different protein composition of A33- and EpCAM-exosomes reflects their release from different cellular surfaces, which may influence their interactions with stromal and immune cells and their contribution to local inflammation [200].

Finally, heat shock proteins 70 (Hsp70) present in CRC-derived exosomes can stimulate the migration and cytolytic activity of NK cells, indicating that exosomal proteins can also have pro-inflammatory and anti-tumor effects, depending on the context [206,207].

## 3. Conclusions and Future Directions

Due to their specific features and important role in tumorigenesis, exosomes represent a new and promising avenue in cancer diagnosis, offering a non-invasive and biomarker-rich tool that could revolutionize early cancer detection and monitoring [151,208,209]. However, challenges of standardization, specificity, sensitivity and clinical validation must be overcome to realize their full potential in clinical practice [150,196,197]. As research progresses, exosomes may become a key component of the future of personalized cancer diagnosis and therapy [209,210].

The integration of AI has significantly improved the analysis and interpretation of exosomal data [211]. Deep learning models, including convolutional neural networks (CNNs) and feature fusion transformers (FFTs), have been successfully applied to exosomal Raman and SERS spectra, enabling accurate early detection of CRC [211,212,213]. Platforms like ChatExosome further utilize large language models and retrieval-augmented generation to provide clinicians with real-time, interpretable, and evidence-based diagnostic recommendations [212].

Despite these advancements, the field faces several biological and technical limitations. The heterogeneity of exosome populations, even from the same cell type, leads to inconsistent cargo concentrations and poses challenges in reproducibility and quantitative analysis [214,215,216]. Moreover, the overlapping content between tumor-derived and normal exosomes complicates the identification of tissue-specific biomarkers [214]. Current isolation techniques also vary in yield and purity, and non-vesicular contamination remains a concern [215]. Lack of standardized protocols for exosome isolation, detection, and profiling continues to hinder large-scale clinical adoption [217]. There is also an absence of universally accepted definitions and classification criteria for exosomes, which further complicates regulatory standardization [218]. To overcome these barriers, novel technologies such as tangential flow filtration, size-exclusion chromatography, and automated microfluidic platforms have been developed to improve consistency, scalability, and clinical applicability [219,220,221]. Moving forward, integration with multiomic profiling, machine learning analytics, and point-of-care biosensor technologies will further increase the clinical impact of exosome-based liquid biopsies [222,223]. However, to ensure broad clinical implementation, interdisciplinary collaboration and validation in multicenter trials will be essential [222,223].

Importantly, several international initiatives have already been undertaken to address the lack of standardization in extracellular vesicle research. The International Society for Extracellular Vesicles (ISEV) has established the Minimal Information for Studies of Extracellular Vesicles (MISEV) guidelines, most recently updated as MISEV2023, which provide recommendations for EV isolation, characterization, and reporting, aiming to improve reproducibility across studies [224]. In parallel, the Lipidomics Standards Initiative (LSI) and related consortia have been developing standardized workflows for lipidomic analyses, including sample handling, extraction, and mass spectrometry-based data reporting [225].

Despite these efforts, standardization remains particularly challenging for exosomal lipidomics and proteomics compared to genetic materials. Lipids and proteins exhibit high structural diversity and dynamic modifications, and are present in low abundance within EVs, making their detection and quantification highly sensitive to methodological variability. Additionally, they are more susceptible to degradation and contamination during isolation procedures, for example by abundant plasma proteins, leading to inconsistencies between studies. In contrast, nucleic acids such as RNA benefit from well-established amplification and sequencing technologies, which improve analytical robustness despite low input material [226,227]. Therefore, the lack of universally accepted protocols represents a major barrier to the clinical translation of lipid- and protein-based exosomal biomarkers in CRC.

Recent clinical studies further support the translational potential of exosome-based diagnostics in colorectal cancer. The EXONERATE clinical trial (NCT05972421) evaluated plasma-derived exosomal protein signatures, including EpCAM, CD133, and glypican-1, as predictive biomarkers of response to anti-EGFR therapy in patients with RAS wild-type metastatic CRC. The results demonstrated high diagnostic accuracy (82%) in distinguishing deep responders from non-responders, outperforming ctDNA-based approaches [228]. These findings highlight the growing clinical applicability of exosome-based liquid biopsy and underscore its potential to improve patient stratification and treatment monitoring in colorectal cancer.

In conclusion, exosomes are poised to revolutionize CRC diagnostics and management. Their molecular diversity, stability, and noninvasive accessibility make them ideal candidates for next-generation biomarkers. As technology matures and clinical barriers are addressed, exosomes will likely become a core component of precision oncology.

## Data Availability

Data sharing is not applicable to this article as no datasets were generated or analyzed during the current study.

## Abbreviations

A33	transmembrane glycoprotein A33
AA	arachidonic acid
ADAMTS13	A disintegrin, and metalloproteinase with thrombospondin motifs 13
AHSG	alpha 2-HS glycoprotein
AI	artificial intelligence
ALA	α-linolenic acid
ANXA1	Annexin A1
AP	adenomatous polyps
APC	adenomatous polyposis coli
ATX	autotaxin
AUC	area under the ROC curve
BRAF	B-Raf proto-oncogene, serine/threonine kinase
CA19-9	carbohydrate antigen 19-9
CCAT1	colon cancer-associated transcript 1
CEA	carcinoembryonic antigen
Cer	ceramides
CIMP	CpG island methylation phenotype
CIN	chromosomal instability
CNNs	convolutional neural networks
CPNE3	copine protein III
CTCs	circulating tumor cells
cfDNA	cell-free DNA
CRC	colorectal cancer
ctDNA	circulating tumor DNA
C3	complement component 3
DCs	dendritic cells
DEPs	differentially expressed proteins
Dkk-3	Dickkopf-3
DLS	dynamic light scattering
EMT	endothelial–mesenchymal transition
EpCAM	epithelial cell adhesion molecule
EPA	eicosapentaenoic acid
ESCRT	endosomal sorting complex required for transport
EVs	extracellular vesicles
FAs	fatty acids
FDA	Food and Drug Administration
FIT	Fecal Immunochemical Test
FN1	fibronectin 1
FS	flexible sigmoidoscopy
gFOBT	guaiac-based Fecal Occult Blood Test
GLA	γ-linolenic acid
GOF	gain of oncogenic functions
GPC1	glipican-1
Her	hereditary non-polyposis colorectal cancer
HexCer	hexosylceramide
HOTAIR	HOX transcript antisense RNA
HP	hyperplastic polyps
HSP90AA1	human stress-inducible 90 kDa heat shock protein alpha
Hsp60	heat shock protein 60
Hsp70	heat shock protein 70
IGF1	insulin-like growth factor I
IGFBP-2	insulin-like growth factor binding protein 2
ILVs	intraluminal vesicles
IRF-2	interferon regulatory factor-2
KRAS	Kirsten rat sarcoma viral oncogene homolog
LA	linoleic acid
LGALS3BP	galectin-binding protein-3
lncRNAs	long non-coding RNAs
LPA	lysophosphatidic acid
MDSCs	myeloid-derived suppressor cells
MMP9	matrix metalloproteinase-9
MSI	microsatellite instability
MTTP	microsomal triglyceride transfer protein
MUFA	monounsaturated fatty acid
MVBs	multivesicular bodies
mt-sDNA	multitargeted stool DNA test
NGS	next-generation sequencing
NK	natural killer
NHP2	NHP2 ribonucleoprotein
NTA	nanoparticle tracking analysis
NSAIDs	non-steroidal anti-inflammatory drugs
OLFM4	olfactomedin 4
OXPHOS	mitochondrial oxidative phosphorylation
PA	phosphatidic acid
PC	phosphatidylcholine
PE	phosphatidylethanolamine
PGE2	prostaglandin E2
PI	phosphatidylinositol
PKM2	pyruvate kinase M2
PS	phosphatidylserine
PUFAs	polyunsaturated fatty acids
p53	tumor protein P53
RPPH1	ribonuclease P RNA component H1
SAMP	small archaeal modifier proteins
S1P	sphingosine-1-phosphate
SM	sphingomyelin
SMAD4	SMAD family member 4
SNAP23	synaptosomal-associated protein 23
SP	sphingolipid
SPE	exosomes purified from serum of patients
TAGL	transgelin
TCF	T cell factor
TEPs	tumor-educated platelets
TME	tumor microenvironment
TOP1	topoisomerase 1
TRIM28	tripartite motif-containing 28
Tregs	regulatory T cells
TP53	tumor protein p53
TUBB3	tubulin β-III
WHO	World Health Organization

## References

[1] Morgan E., Arnold M., Gini A., Lorenzoni V., Cabasag C.J., Laversanne M., Vignat J., Ferlay J., Murphy N., Bray F. (2023). Global Burden of Colorectal Cancer in 2020 and 2040: Incidence and Mortality Estimates from GLOBOCAN. Gut.

[2] Li C., Zheng H., Jia H., Huang D., Gu W., Cai S., Zhu J. (2019). Prognosis of Three Histological Subtypes of Colorectal Adenocarcinoma: A Retrospective Analysis of 8005 Chinese Patients. Cancer Med..

[3] Hultcrantz R. (2021). Aspects of Colorectal Cancer Screening, Methods, Age and Gender. J. Intern. Med..

[4] Stoffel E.M., Murphy C.C. (2020). Epidemiology and Mechanisms of the Increasing Incidence of Colon and Rectal Cancers in Young Adults. Gastroenterology.

[5] Hossain M.S., Karuniawati H., Jairoun A.A., Urbi Z., Ooi D.J., John A., Lim Y.C., Kibria K.M.K., Mohiuddin A.K.M., Ming L.C. (2022). Colorectal Cancer: A Review of Carcinogenesis, Global Epidemiology, Current Challenges, Risk Factors, Preventive and Treatment Strategies. Cancers.

[6] Zhang Y., Wang Y., Zhang B., Li P., Zhao Y. (2023). Methods and Biomarkers for Early Detection, Prediction, and Diagnosis of Colorectal Cancer. Biomed. Pharmacother..

[7] Balacescu O., Sur D., Cainap C., Visan S., Cruceriu D., Manzat-Saplacan R., Muresan M.-S., Balacescu L., Lisencu C., Irimie A. (2018). The Impact of miRNA in Colorectal Cancer Progression and Its Liver Metastases. Int. J. Mol. Sci..

[8] Huang D., Sun W., Zhou Y., Li P., Chen F., Chen H., Xia D., Xu E., Lai M., Wu Y. (2018). Mutations of Key Driver Genes in Colorectal Cancer Progression and Metastasis. Cancer Metastasis Rev..

[9] Dow L.E., O’Rourke K.P., Simon J., Tschaharganeh D.F., van Es J.H., Clevers H., Lowe S.W. (2015). Apc Restoration Promotes Cellular Differentiation and Reestablishes Crypt Homeostasis in Colorectal Cancer. Cell.

[10] Eklöf V., Wikberg M.L., Edin S., Dahlin A.M., Jonsson B.-A., Öberg Å., Rutegård J., Palmqvist R. (2013). The Prognostic Role of KRAS, BRAF, PIK3CA and PTEN in Colorectal Cancer. Br. J. Cancer.

[11] Vogelstein B., Papadopoulos N., Velculescu V.E., Zhou S., Diaz L.A., Kinzler K.W. (2013). Cancer Genome Landscapes. Science.

[12] Shibata D., Reale M.A., Lavin P., Silverman M., Fearon E.R., Steele G., Jessup J.M., Loda M., Summerhayes I.C. (1996). The DCC Protein and Prognosis in Colorectal Cancer. N. Engl. J. Med..

[13] Duman-Scheel M. (2012). Deleted in Colorectal Cancer (DCC) Pathfinding: Axon Guidance Gene Finally Turned Tumor Suppressor. Curr. Drug Targets.

[14] Inokuchi K., Yamaguchi H., Hanawa H., Tanosaki S., Nakamura K., Tarusawa M., Miyake K., Shimada T., Dan K. (2002). Loss of DCC Gene Expression Is of Prognostic Importance in Acute Myelogenous Leukemia. Clin. Cancer Res..

[15] Tamraz M., Al Ghossaini N., Temraz S. (2024). Optimization of Colorectal Cancer Screening Strategies: New Insights. World J. Gastroenterol..

[16] Lieberman D.A., Weiss D.G., Veterans Affairs Cooperative Study Group 380 (2001). One-Time Screening for Colorectal Cancer with Combined Fecal Occult-Blood Testing and Examination of the Distal Colon. N. Engl. J. Med..

[17] Quintero E., Castells A., Bujanda L., Cubiella J., Salas D., Lanas Á., Andreu M., Carballo F., Morillas J.D., Hernández C. (2012). Colonoscopy versus Fecal Immunochemical Testing in Colorectal-Cancer Screening. N. Engl. J. Med..

[18] Brenner H., Stock C., Hoffmeister M. (2014). Effect of Screening Sigmoidoscopy and Screening Colonoscopy on Colorectal Cancer Incidence and Mortality: Systematic Review and Meta-Analysis of Randomised Controlled Trials and Observational Studies. BMJ.

[19] Cusumano V.T., May F.P. (2020). Making FIT Count: Maximizing Appropriate Use of the Fecal Immunochemical Test for Colorectal Cancer Screening Programs. J. Gen. Intern. Med..

[20] Rabeneck L., Rumble R.B., Thompson F., Mills M., Oleschuk C., Whibley A., Messersmith H., Lewis N. (2012). Fecal Immunochemical Tests Compared with Guaiac Fecal Occult Blood Tests for Population-Based Colorectal Cancer Screening. Can. J. Gastroenterol..

[21] Imperiale T.F., Ransohoff D.F., Itzkowitz S.H., Levin T.R., Lavin P., Lidgard G.P., Ahlquist D.A., Berger B.M. (2014). Multitarget Stool DNA Testing for Colorectal-Cancer Screening. N. Engl. J. Med..

[22] Marcuello M., Vymetalkova V., Neves R.P.L., Duran-Sanchon S., Vedeld H.M., Tham E., van Dalum G., Flügen G., Garcia-Barberan V., Fijneman R.J. (2019). Circulating Biomarkers for Early Detection and Clinical Management of Colorectal Cancer. Mol. Asp. Med..

[23] Xu M., Qi P., Du X. (2014). Long Non-Coding RNAs in Colorectal Cancer: Implications for Pathogenesis and Clinical Application. Mod. Pathol..

[24] Church T.R., Wandell M., Lofton-Day C., Mongin S.J., Burger M., Payne S.R., Castaños-Vélez E., Blumenstein B.A., Rösch T., Osborn N. (2014). Prospective Evaluation of Methylated SEPT9 in Plasma for Detection of Asymptomatic Colorectal Cancer. Gut.

[25] Hung C.-S., Huang C.-Y., Lee C.-H., Chen W.-Y., Huang M.-T., Wei P.-L., Chang Y.-J. (2017). IGFBP2 Plays an Important Role in Heat Shock Protein 27-Mediated Cancer Progression and Metastasis. Oncotarget.

[26] Peabody J., Rahim A., Wilcox B., McGehee C., Estigarribia E., Paculdo D., Arzadon A., Fugaro S., Tran M., Spitzer G. (2019). Clinical Utility of a Blood-Based Protein Assay on Diagnostic Colonoscopy Referrals for Elevated-Risk Colorectal Cancer Patients in Primary Care. Am. J. Clin. Oncol..

[27] Ullah I., Yang L., Yin F.-T., Sun Y., Li X.-H., Li J., Wang X.-J. (2022). Multi-Omics Approaches in Colorectal Cancer Screening and Diagnosis, Recent Updates and Future Perspectives. Cancers.

[28] Paraskar G., Bhattacharya S., Kuttiappan A. (2025). The Role of Proteomics and Genomics in the Development of Colorectal Cancer Diagnostic Tools and Potential New Treatments. ACS Pharmacol. Transl. Sci..

[29] Addissouky T.A., Sayed I.E.T.E., Ali M.M.A., Alubiady M.H.S., Wang Y. (2024). Precision Medicine and Immunotherapy Advances Transforming Colorectal Cancer Treatment. J. Cancer Biol..

[30] Goyal H., Mann R., Gandhi Z., Perisetti A., Ali A., Aman Ali K., Sharma N., Saligram S., Tharian B., Inamdar S. (2020). Scope of Artificial Intelligence in Screening and Diagnosis of Colorectal Cancer. J. Clin. Med..

[31] Tokutake K., Morelos-Gomez A., Hoshi K., Katouda M., Tejima S., Endo M. (2023). Artificial Intelligence for the Prevention and Prediction of Colorectal Neoplasms. J. Transl. Med..

[32] Xu H., Tang R.S.Y., Lam T.Y.T., Zhao G., Lau J.Y.W., Liu Y., Wu Q., Rong L., Xu W., Li X. (2023). Artificial Intelligence-Assisted Colonoscopy for Colorectal Cancer Screening: A Multicenter Randomized Controlled Trial. Clin. Gastroenterol. Hepatol..

[33] Baxter N.N., Goldwasser M.A., Paszat L.F., Saskin R., Urbach D.R., Rabeneck L. (2009). Association of Colonoscopy and Death from Colorectal Cancer. Ann. Intern. Med..

[34] Manser C.N., Bachmann L.M., Brunner J., Hunold F., Bauerfeind P., Marbet U.A. (2012). Colonoscopy Screening Markedly Reduces the Occurrence of Colon Carcinomas and Carcinoma-Related Death: A Closed Cohort Study. Gastrointest. Endosc..

[35] Kahi C.J., Imperiale T.F., Juliar B.E., Rex D.K. (2009). Effect of Screening Colonoscopy on Colorectal Cancer Incidence and Mortality. Clin. Gastroenterol. Hepatol..

[36] Nishihara R., Wu K., Lochhead P., Morikawa T., Liao X., Qian Z.R., Inamura K., Kim S.A., Kuchiba A., Yamauchi M. (2013). Long-Term Colorectal-Cancer Incidence and Mortality after Lower Endoscopy. N. Engl. J. Med..

[37] Ransohoff D.F. (2009). How Much Does Colonoscopy Reduce Colon Cancer Mortality?. Ann. Intern. Med..

[38] Singh H., Nugent Z., Demers A.A., Kliewer E.V., Mahmud S.M., Bernstein C.N. (2010). The Reduction in Colorectal Cancer Mortality After Colonoscopy Varies by Site of the Cancer. Gastroenterology.

[39] Sandler R.S. (2010). Colonoscopy and Colorectal Cancer Mortality: Strong Beliefs or Strong Facts?. Am. J. Gastroenterol..

[40] Neugut A.I., Lebwohl B. (2010). Colonoscopy vs Sigmoidoscopy Screening: Getting It Right. JAMA.

[41] Jørgensen O.D., Kronborg O., Fenger C., Rasmussen M. (2007). Influence of Long-Term Colonoscopic Surveillance on Incidence of Colorectal Cancer and Death from the Disease in Patients with Precursors (Adenomas). Acta Oncol..

[42] Brenner H., Chang-Claude J., Seiler C.M., Rickert A., Hoffmeister M. (2011). Protection From Colorectal Cancer After Colonoscopy. Ann. Intern. Med..

[43] Jarbol D.E., Kragstrup J., Stovring H., Havelund T., de Muckadell O.B.S. (2006). Proton Pump Inhibitor or Testing for Helicobacter Pylori as the First Step for Patients Presenting with Dyspepsia? A Cluster-Randomized Trial. Off. J. Am. Coll. Gastroenterol..

[44] Lieberman D. (2006). A Call to Action--Measuring the Quality of Colonoscopy. N. Engl. J. Med..

[45] Kaminski M.F., Regula J., Kraszewska E., Polkowski M., Wojciechowska U., Didkowska J., Zwierko M., Rupinski M., Nowacki M.P., Butruk E. (2010). Quality Indicators for Colonoscopy and the Risk of Interval Cancer. N. Engl. J. Med..

[46] Hoff G., Grotmol T., Skovlund E., Bretthauer M., Norwegian Colorectal Cancer Prevention Study Group (2009). Risk of Colorectal Cancer Seven Years after Flexible Sigmoidoscopy Screening: Randomised Controlled Trial. BMJ.

[47] Segnan N., Armaroli P., Bonelli L., Risio M., Sciallero S., Zappa M., Andreoni B., Arrigoni A., Bisanti L., Casella C. (2011). Once-Only Sigmoidoscopy in Colorectal Cancer Screening: Follow-up Findings of the Italian Randomized Controlled Trial—SCORE. J. Natl. Cancer Inst..

[48] Allison J.E., Tekawa I.S., Ransom L.J., Adrain A.L. (1996). A Comparison of Fecal Occult-Blood Tests for Colorectal-Cancer Screening. N. Engl. J. Med..

[49] Cooper R.E., Hutchinson E.K., Izzi J.M. (2020). Evaluation of the Guaiac Fecal Occult Blood Test for Detection of Gastrointestinal Bleeding in the Rhesus Macaque (*Macaca mulatta*). J. Med. Primatol..

[50] Diedrich L., Brinkmann M., Dreier M., Rossol S., Schramm W., Krauth C. (2023). Is There a Place for Sigmoidoscopy in Colorectal Cancer Screening? A Systematic Review and Critical Appraisal of Cost-Effectiveness Models. PLoS ONE.

[51] Rabeneck L., Zwaal C., Goodman J.H., Mai V., Zamkanei M. (2008). Cancer Care Ontario Guaiac Fecal Occult Blood Test (FOBT) Laboratory Standards: Evidentiary Base and Recommendations. Clin. Biochem..

[52] Hewitson P., Glasziou P., Watson E., Towler B., Irwig L. (2008). Cochrane Systematic Review of Colorectal Cancer Screening Using the Fecal Occult Blood Test (Hemoccult): An Update. Off. J. Am. Coll. Gastroenterol..

[53] Mandel J.S., Bond J.H., Church T.R., Snover D.C., Bradley G.M., Schuman L.M., Ederer F. (1993). Reducing Mortality from Colorectal Cancer by Screening for Fecal Occult Blood. N. Engl. J. Med..

[54] Fraser C.G., Matthew C.M., Mowat N.A.G., Wilson J.A., Carey F.A., Steele R.J.C. (2006). Immunochemical Testing of Individuals Positive for Guaiac Faecal Occult Blood Test in a Screening Programme for Colorectal Cancer: An Observational Study. Lancet Oncol..

[55] Huang J., Chen M., Chan V.C.W., Liu X., Zhong C., Lin J., Hang J., Zhong C.C., Yuan J., Wong M.C.S. (2025). The Cost-Effectiveness of a Multi-Target Stool DNA-Based Screening (COLOTECT), FIT, Colonoscopy and No Screening for Colorectal Cancer. Cancer Rep..

[56] van Rossum L.G., van Rijn A.F., Laheij R.J., van Oijen M.G., Fockens P., van Krieken H.H., Verbeek A.L., Jansen J.B., Dekker E. (2008). Random Comparison of Guaiac and Immunochemical Fecal Occult Blood Tests for Colorectal Cancer in a Screening Population. Gastroenterology.

[57] D’Souza N., Georgiou Delisle T., Chen M., Benton S., Abulafi M. (2021). Faecal Immunochemical Test Is Superior to Symptoms in Predicting Pathology in Patients with Suspected Colorectal Cancer Symptoms Referred on a 2WW Pathway: A Diagnostic Accuracy Study. Gut.

[58] Berger B.M., Levin B., Hilsden R.J. (2016). Multitarget Stool DNA for Colorectal Cancer Screening: A Review and Commentary on the United States Preventive Services Draft Guidelines. World J. Gastrointest. Oncol..

[59] Lopes S.R., Martins C., Santos I.C., Teixeira M., Gamito É., Alves A.L. (2024). Colorectal Cancer Screening: A Review of Current Knowledge and Progress in Research. World J. Gastrointest. Oncol..

[60] Dolatkhah R., Dastgiri S., Jafarabadi M.A., Abdolahi H.M., Somi M.H. (2022). Diagnostic accuracy of multitarget stool DNA testing for colorectal cancer screening: A systematic review and meta-analysis. Gastroenterol. Hepatol..

[61] Ladabaum U., Mannalithara A. (2016). Comparative Effectiveness and Cost Effectiveness of a Multitarget Stool DNA Test to Screen for Colorectal Neoplasia. Gastroenterology.

[62] Eckmann J.D., Ebner D.W., Kisiel J.B. (2020). Multi-Target Stool DNA Testing for Colorectal Cancer Screening: Emerging Learning on Real-World Performance. Curr. Treat. Options Gastroenterol..

[63] Kisiel J.B., Limburg P.J. (2020). Colorectal Cancer Screening With the Multitarget Stool DNA Test. Am. J. Gastroenterol..

[64] Naber S.K., Knudsen A.B., Zauber A.G., Rutter C.M., Fischer S.E., Pabiniak C.J., Soto B., Kuntz K.M., Lansdorp-Vogelaar I. (2019). Cost-Effectiveness of a Multitarget Stool DNA Test for Colorectal Cancer Screening of Medicare Beneficiaries. PLoS ONE.

[65] Coronado G.D., Jenkins C.L., Shuster E., Johnson C., Amy D., Cook J., Sahnow S., Zepp J.M., Mummadi R. (2024). Blood-Based Colorectal Cancer Screening in an Integrated Health System: A Randomised Trial of Patient Adherence. Gut.

[66] Lofton-Day C., Model F., DeVos T., Tetzner R., Distler J., Schuster M., Song X., Lesche R., Liebenberg V., Ebert M. (2008). DNA Methylation Biomarkers for Blood-Based Colorectal Cancer Screening. Clin. Chem..

[67] Zapka J., Klabunde C.N., Taplin S., Yuan G., Ransohoff D., Kobrin S. (2012). Screening Colonoscopy in the US: Attitudes and Practices of Primary Care Physicians. J. Gen. Intern. Med..

[68] Juul F.E., Cross A.J., Schoen R.E., Senore C., Pinsky P.F., Miller E.A., Segnan N., Wooldrage K., Wieszczy-Szczepanik P., Armaroli P. (2024). Effectiveness of Colonoscopy Screening vs Sigmoidoscopy Screening in Colorectal Cancer. JAMA Netw. Open.

[69] Ahlquist D.A. (2019). Stool-Based Tests Vs Screening Colonoscopy for the Detection of Colorectal Cancer. Gastroenterol. Hepatol..

[70] Niikura R., Nagata N., Yamada A., Honda T., Hasatani K., Ishii N., Shiratori Y., Doyama H., Nishida T., Sumiyoshi T. (2020). Efficacy and Safety of Early vs Elective Colonoscopy for Acute Lower Gastrointestinal Bleeding. Gastroenterology.

[71] Ibáñez J., Vanaclocha-Espí M., Pérez-Sanz E., Valverde M.J., Sáez-Lloret I., Molina-Barceló A., Salas D. (2018). Severe Complications in Colorectal Cancer Screening Colonoscopies in the Valencian Community. Gastroenterol. Hepatol..

[72] Fisher D.A., Maple J.T., Ben-Menachem T., Cash B.D., Decker G.A., Early D.S., Evans J.A., Fanelli R.D., Fukami N., Hwang J.H. (2011). Complications of Colonoscopy. Gastrointest. Endosc..

[73] Harlid S., Gunter M.J., Van Guelpen B. (2021). Risk-Predictive and Diagnostic Biomarkers for Colorectal Cancer; a Systematic Review of Studies Using Pre-Diagnostic Blood Samples Collected in Prospective Cohorts and Screening Settings. Cancers.

[74] Clark-Langone K.M., Wu J.Y., Sangli C., Chen A., Snable J.L., Nguyen A., Hackett J.R., Baker J., Yothers G., Kim C. (2007). Biomarker Discovery for Colon Cancer Using a 761 Gene RT-PCR Assay. BMC Genom..

[75] Hilal G., Reitzel R., Al Hamal Z., Chaftari A.-M., Al Wohoush I., Jiang Y., Hachem R., Raad I.I. (2017). Novel Plasma Telomerase Detection Method to Improve Cancer Diagnostic Assessment. PLoS ONE.

[76] Danese E., Montagnana M., Minicozzi A.M., Matteis G.D., Scudo G., Salvagno G.L., Cordiano C., Lippi G., Guidi G.C. (2010). Real-Time Polymerase Chain Reaction Quantification of Free DNA in Serum of Patients with Polyps and Colorectal Cancers. Clin. Chem. Lab. Med..

[77] Ciombor K.K., Haraldsdottir S., Goldberg R.M. (2014). How Can Next-Generation Sequencing (Genomics) Help Us in Treating Colorectal Cancer?. Curr. Color. Cancer Rep..

[78] Kim R.Y., Xu H., Myllykangas S., Ji H. (2011). Genetic-Based Biomarkers and next-Generation Sequencing: The Future of Personalized Care in Colorectal Cancer. Pers. Med..

[79] Morasso C., Daveri E., Bonizzi A., Truffi M., Colombo F., Danelli P., Albasini S., Rivoltini L., Mazzucchelli S., Sorrentino L. (2024). Raman Spectroscopy on Dried Blood Plasma Allows Diagnosis and Monitoring of Colorectal Cancer. MedComm.

[80] Bessa X., Vidal J., Balboa J.C., Márquez C., Duenwald S., He Y., Raymond V., Faull I., Burón A., Álvarez-Urturi C. (2023). High Accuracy of a Blood ctDNA-Based Multimodal Test to Detect Colorectal Cancer. Ann. Oncol..

[81] Ladabaum U., Mannalithara A., Weng Y., Schoen R.E., Dominitz J.A., Desai M., Lieberman D. (2024). Comparative Effectiveness and Cost-Effectiveness of Colorectal Cancer Screening With Blood-Based Biomarkers (Liquid Biopsy) vs Fecal Tests or Colonoscopy. Gastroenterology.

[82] Rex D.K., Johnson D.A., Anderson J.C., Schoenfeld P.S., Burke C.A., Inadomi J.M. (2009). American College of Gastroenterology Guidelines for Colorectal Cancer Screening 2008. Off. J. Am. Coll. Gastroenterol..

[83] Mansour H. (2014). Cell-Free Nucleic Acids as Noninvasive Biomarkers for Colorectal Cancer Detection. Front. Genet..

[84] Danese E., Montagnana M., Lippi G. (2019). Circulating Molecular Biomarkers for Screening or Early Diagnosis of Colorectal Cancer: Which Is Ready for Prime Time?. Ann. Transl. Med..

[85] Wills B., Gorse E., Lee V. (2018). Role of Liquid Biopsies in Colorectal Cancer. Curr. Probl. Cancer.

[86] Normanno N., Cervantes A., Ciardiello F., Luca A.D., Pinto C. (2018). The Liquid Biopsy in the Management of Colorectal Cancer Patients: Current Applications and Future Scenarios. Cancer Treat. Rev..

[87] Vacante M., Ciuni R., Basile F., Biondi A. (2020). The Liquid Biopsy in the Management of Colorectal Cancer: An Overview. Biomedicines.

[88] Alix-Panabières C., Pantel K. (2014). Challenges in Circulating Tumour Cell Research. Nat. Rev. Cancer.

[89] Su W., Yu H., Jiang L., Chen W., Li H., Qin J. (2019). Integrated Microfluidic Device for Enrichment and Identification of Circulating Tumor Cells from the Blood of Patients with Colorectal Cancer. Dis. Markers.

[90] Wu J., Li Z., Zou J., Li L., Cui N., Hao T., Yi K., Yang J., Wu Y. (2022). A Meta-Analysis of the Value of Circulating Tumor Cells in Monitoring Postoperative Recurrence and Metastasis of Colorectal Cancer. PLoS ONE.

[91] Thierry A.R., El Messaoudi S., Gahan P.B., Anker P., Stroun M. (2016). Origins, Structures, and Functions of Circulating DNA in Oncology. Cancer Metastasis Rev..

[92] Stroun M., Lyautey J., Lederrey C., Olson-Sand A., Anker P. (2001). About the Possible Origin and Mechanism of Circulating DNA: Apoptosis and Active DNA Release. Clin. Chim. Acta.

[93] Osumi H., Shinozaki E., Yamaguchi K., Zembutsu H. (2019). Clinical Utility of Circulating Tumor DNA for Colorectal Cancer. Cancer Sci..

[94] Liu H.-C., Han D.-S., Hsu C.-C., Wang J.-S. (2021). Circulating MicroRNA-486 and MicroRNA-146a Serve as Potential Biomarkers of Sarcopenia in the Older Adults. BMC Geriatr..

[95] Schirripa M., Borelli B., D’Aurizio R., Lubrano S., Cremolini C., Zucchelli G., Antoniotti C., Marmorino F., Prete A.A., Murgioni S. (2019). Early Modifications of Circulating microRNAs Levels in Metastatic Colorectal Cancer Patients Treated with Regorafenib. Pharmacogenomics J..

[96] Van Roosbroeck K., Calin G.A., Croce C.M., Fisher P.B. (2017). Chapter Four—Cancer Hallmarks and MicroRNAs: The Therapeutic Connection. Advances in Cancer Research.

[97] McDonald J.S., Milosevic D., Reddi H.V., Grebe S.K., Algeciras-Schimnich A. (2011). Analysis of Circulating MicroRNA: Preanalytical and Analytical Challenges. Clin. Chem..

[98] Coleman D., Kuwada S. (2024). miRNA as a Biomarker for the Early Detection of Colorectal Cancer. Genes..

[99] Wang H., Peng R., Wang J., Qin Z., Xue L. (2018). Circulating microRNAs as Potential Cancer Biomarkers: The Advantage and Disadvantage. Clin. Epigenetics.

[100] Cheng H.H., Yi H.S., Kim Y., Kroh E.M., Chien J.W., Eaton K.D., Goodman M.T., Tait J.F., Tewari M., Pritchard C.C. (2013). Plasma Processing Conditions Substantially Influence Circulating microRNA Biomarker Levels. PLoS ONE.

[101] Healy N.A., Heneghan H.M., Miller N., Osborne C.K., Schiff R., Kerin M.J. (2012). Systemic Mirnas as Potential Biomarkers for Malignancy. Int. J. Cancer.

[102] Høye E., Fromm B., Böttger P.H.M., Domanska D., Torgunrud A., Lund-Andersen C., Abrahamsen T.W., Fretland Å.A., Dagenborg V.J., Lorenz S. (2022). A Comprehensive Framework for Analysis of microRNA Sequencing Data in Metastatic Colorectal Cancer. NAR Cancer.

[103] Zhao W., Song M., Zhang J., Kuerban M., Wang H. (2015). Combined Identification of Long Non-Coding RNA CCAT1 and HOTAIR in Serum as an Effective Screening for Colorectal Carcinoma. Int. J. Clin. Exp. Pathol..

[104] Sole C., Arnaiz E., Manterola L., Otaegui D., Lawrie C.H. (2019). The Circulating Transcriptome as a Source of Cancer Liquid Biopsy Biomarkers. Semin. Cancer Biol..

[105] Shi T., Gao G., Cao Y. (2016). Long Noncoding RNAs as Novel Biomarkers Have a Promising Future in Cancer Diagnostics. Dis. Markers.

[106] Benes V., Castoldi M. (2010). Expression Profiling of microRNA Using Real-Time Quantitative PCR, How to Use It and What Is Available. Methods.

[107] Yu H., Rohan T. (2000). Role of the Insulin-Like Growth Factor Family in Cancer Development and Progression. J. Natl. Cancer Inst..

[108] Renehan A.G., Jones J., Potten C.S., Shalet S.M., O’Dwyer S.T. (2000). Elevated Serum Insulin-like Growth Factor (IGF)-II and IGF Binding Protein-2 in Patients with Colorectal Cancer. Br. J. Cancer.

[109] Hardt P.D., Ewald N. (2008). Tumor M2 Pyruvate Kinase: A Tumor Marker and Its Clinical Application in Gastrointestinal Malignancy. Expert Rev. Mol. Diagn..

[110] Li R., Liu J., Xue H., Huang G. (2012). Diagnostic Value of Fecal Tumor M2-Pyruvate Kinase for CRC Screening: A Systematic Review and Meta-Analysis. Int. J. Cancer.

[111] Sithambaram S., Hilmi I., Goh K.-L. (2015). The Diagnostic Accuracy of the M2 Pyruvate Kinase Quick Stool Test- A Rapid Office Based Assay Test for the Detection of Colorectal Cancer. PLoS ONE.

[112] Niehrs C. (2006). Function and Biological Roles of the Dickkopf Family of Wnt Modulators. Oncogene.

[113] Krupnik V.E., Sharp J.D., Jiang C., Robison K., Chickering T.W., Amaravadi L., Brown D.E., Guyot D., Mays G., Leiby K. (1999). Functional and Structural Diversity of the Human Dickkopf Gene Family. Gene.

[114] Sato H., Suzuki H., Toyota M., Nojima M., Maruyama R., Sasaki S., Takagi H., Sogabe Y., Sasaki Y., Idogawa M. (2007). Frequent Epigenetic Inactivation of DICKKOPF Family Genes in Human Gastrointestinal Tumors. Carcinogenesis.

[115] Fung K.Y.C., Tabor B., Buckley M.J., Priebe I.K., Purins L., Pompeia C., Brierley G.V., Lockett T., Gibbs P., Tie J. (2015). Blood-Based Protein Biomarker Panel for the Detection of Colorectal Cancer. PLoS ONE.

[116] Jeppesen D.K., Fenix A.M., Franklin J.L., Higginbotham J.N., Zhang Q., Zimmerman L.J., Liebler D.C., Ping J., Liu Q., Evans R. (2019). Reassessment of Exosome Composition. Cell.

[117] Kalluri R. (2016). The Biology and Function of Exosomes in Cancer. J. Clin. Invest..

[118] Rajagopal C., Harikumar K.B. (2018). The Origin and Functions of Exosomes in Cancer. Front. Oncol..

[119] Huda M.N., Nafiujjaman M., Deaguero I.G., Okonkwo J., Hill M.L., Kim T., Nurunnabi M. (2021). Potential Use of Exosomes as Diagnostic Biomarkers and in Targeted Drug Delivery: Progress in Clinical and Preclinical Applications. ACS Biomater. Sci. Eng..

[120] Rahmati S., Shojaei F., Shojaeian A., Rezakhani L., Dehkordi M.B. (2020). An Overview of Current Knowledge in Biological Functions and Potential Theragnostic Applications of Exosomes. Chem. Phys. Lipids.

[121] Jan A.T., Rahman S., Khan S., Tasduq S.A., Choi I. (2019). Biology, Pathophysiological Role, and Clinical Implications of Exosomes: A Critical Appraisal. Cells.

[122] Sokolova V., Ludwig A.-K., Hornung S., Rotan O., Horn P.A., Epple M., Giebel B. (2011). Characterisation of Exosomes Derived from Human Cells by Nanoparticle Tracking Analysis and Scanning Electron Microscopy. Colloids Surf. B Biointerfaces.

[123] Li M., Li S., Du C., Zhang Y., Li Y., Chu L., Han X., Galons H., Zhang Y., Sun H. (2020). Exosomes from Different Cells: Characteristics, Modifications, and Therapeutic Applications. Eur. J. Med. Chem..

[124] Braicu C., Tomuleasa C., Monroig P., Cucuianu A., Berindan-Neagoe I., Calin G.A. (2015). Exosomes as Divine Messengers: Are They the Hermes of Modern Molecular Oncology?. Cell Death Differ..

[125] Pocsfalvi G., Stanly C., Vilasi A., Fiume I., Capasso G., Turiák L., Buzas E.I., Vékey K. (2016). Mass Spectrometry of Extracellular Vesicles. Mass. Spectrom. Rev..

[126] Greening D.W., Xu R., Gopal S.K., Rai A., Simpson R.J. (2017). Proteomic Insights into Extracellular Vesicle Biology—Defining Exosomes and Shed Microvesicles. Expert Rev. Proteom..

[127] Han Q.-F., Li W.-J., Hu K.-S., Gao J., Zhai W.-L., Yang J.-H., Zhang S.-J. (2022). Exosome Biogenesis: Machinery, Regulation, and Therapeutic Implications in Cancer. Mol. Cancer.

[128] Arya S.B., Collie S.P., Parent C.A. (2024). The Ins-and-Outs of Exosome Biogenesis, Secretion, and Internalization. Trends Cell Biol..

[129] Yan W., Jiang S. (2020). Immune Cell-Derived Exosomes in the Cancer-Immunity Cycle. Trends Cancer.

[130] Mannavola F., Salerno T., Passarelli A., Tucci M., Internò V., Silvestris F. (2019). Revisiting the Role of Exosomes in Colorectal Cancer: Where Are We Now?. Front. Oncol..

[131] Bhome R., Goh R.W., Bullock M.D., Pillar N., Thirdborough S.M., Mellone M., Mirnezami R., Galea D., Veselkov K., Gu Q. (2017). Exosomal microRNAs Derived from Colorectal Cancer-Associated Fibroblasts: Role in Driving Cancer Progression. Aging.

[132] Wadhonkar K., Singh N., Heralde F.M., Parihar S.P., Hirani N., Baig M.S. (2023). Exosome-Derived miRNAs Regulate Macrophage-Colorectal Cancer Cell Cross-Talk during Aggressive Tumor Development. Color. Cancer.

[133] Zhang Y., Tang S., Gao Y., Lu Z., Yang Y., Chen J., Li T. (2024). Application of Exosomal miRNA Mediated Macrophage Polarization in Colorectal Cancer: Current Progress and Challenges. Oncol. Res..

[134] Di Bella M.A. (2022). Overview and Update on Extracellular Vesicles: Considerations on Exosomes and Their Application in Modern Medicine. Biology.

[135] Kalluri R., LeBleu V.S. (2020). The Biology, Function, and Biomedical Applications of Exosomes. Science.

[136] Qiao L., Hu S., Huang K., Su T., Li Z., Vandergriff A., Cores J., Dinh P.-U., Allen T., Shen D. (2020). Tumor Cell-Derived Exosomes Home to Their Cells of Origin and Can Be Used as Trojan Horses to Deliver Cancer Drugs. Theranostics.

[137] Wang X., Tian L., Lu J., Ng I.O.-L. (2022). Exosomes and Cancer—Diagnostic and Prognostic Biomarkers and Therapeutic Vehicle. Oncogenesis.

[138] Cappello F., Fais S. (2022). Extracellular Vesicles in Cancer Pros and Cons: The Importance of the Evidence-Based Medicine. Semin. Cancer Biol..

[139] Jayaseelan V.P. (2020). Emerging Role of Exosomes as Promising Diagnostic Tool for Cancer. Cancer Gene Ther..

[140] Cheshomi H., Matin M.M. (2019). Exosomes and Their Importance in Metastasis, Diagnosis, and Therapy of Colorectal Cancer. J. Cell. Biochem..

[141] Huber V., Fais S., Iero M., Lugini L., Canese P., Squarcina P., Zaccheddu A., Colone M., Arancia G., Gentile M. (2005). Human Colorectal Cancer Cells Induce T-Cell Death Through Release of Proapoptotic Microvesicles: Role in Immune Escape. Gastroenterology.

[142] Lou J., Huang J., Dai X., Xie Y., Dong M., Chen B., Zhao J., Zhou H., Zhou B., Yu H. (2017). Knockdown of Tetraspanin 13 Inhibits Proliferation of Colorectal Cancer Cells. Int. J. Clin. Exp. Med..

[143] Ling H., Spizzo R., Atlasi Y., Nicoloso M., Shimizu M., Redis R.S., Nishida N., Gafà R., Song J., Guo Z. (2013). CCAT2, a Novel Noncoding RNA Mapping to 8q24, Underlies Metastatic Progression and Chromosomal Instability in Colon Cancer. Genome Res..

[144] Wang X., Ding X., Nan L., Wang Y., Wang J., Yan Z., Zhang W., Sun J., Zhu W., Ni B. (2015). Investigation of the Roles of Exosomes in Colorectal Cancer Liver Metastasis. Oncol. Rep..

[145] Lugini L., Valtieri M., Federici C., Cecchetti S., Meschini S., Condello M., Signore M., Fais S. (2016). Exosomes from Human Colorectal Cancer Induce a Tumor-like Behavior in Colonic Mesenchymal Stromal Cells. Oncotarget.

[146] Ju J. (2011). Implications of miRNAs in Colorectal Cancer Chemoresistance. Int. Drug Discov..

[147] Greening D.W., Gopal S.K., Mathias R.A., Liu L., Sheng J., Zhu H.-J., Simpson R.J. (2015). Emerging Roles of Exosomes during Epithelial–Mesenchymal Transition and Cancer Progression. Semin. Cell Dev. Biol..

[148] Mulvey H.E., Chang A., Adler J., Del Tatto M., Perez K., Quesenberry P.J., Chatterjee D. (2015). Extracellular Vesicle-Mediated Phenotype Switching in Malignant and Non-Malignant Colon Cells. BMC Cancer.

[149] Rizk N.I., Abulsoud A.I., Kamal M.M., Kassem D.H., Hamdy N.M. (2022). Exosomal-Long Non-Coding RNAs Journey in Colorectal Cancer: Evil and Goodness Faces of Key Players. Life Sci..

[150] Wang Z., Wang Q., Qin F., Chen J. (2024). Exosomes: A Promising Avenue for Cancer Diagnosis beyond Treatment. Front. Cell Dev. Biol..

[151] Andre M., Caobi A., Miles J.S., Vashist A., Ruiz M.A., Raymond A.D. (2024). Diagnostic Potential of Exosomal Extracellular Vesicles in Oncology. BMC Cancer.

[152] Germain N., Dhayer M., Boileau M., Fovez Q., Kluza J., Marchetti P. (2020). Lipid Metabolism and Resistance to Anticancer Treatment. Biology.

[153] Elsherbini A., Bieberich E., Chalfant C.E., Fisher P.B. (2018). Chapter Five—Ceramide and Exosomes: A Novel Target in Cancer Biology and Therapy. Advances in Cancer Research.

[154] Rice G.E. (1998). Secretory Phospholipases and Membrane Polishing. Placenta.

[155] Skotland T., Hessvik N.P., Sandvig K., Llorente A. (2019). Exosomal Lipid Composition and the Role of Ether Lipids and Phosphoinositides in Exosome Biology. J. Lipid Res..

[156] Kopecka J., Trouillas P., Gašparović A.Č., Gazzano E., Assaraf Y.G., Riganti C. (2020). Phospholipids and Cholesterol: Inducers of Cancer Multidrug Resistance and Therapeutic Targets. Drug Resist. Updates.

[157] Taniguchi M., Okazaki T. (2014). The Role of Sphingomyelin and Sphingomyelin Synthases in Cell Death, Proliferation and Migration—From Cell and Animal Models to Human Disorders. Biochim. Et Biophys. Acta Mol. Cell Biol. Lipids.

[158] Beloribi-Djefaflia S., Siret C., Lombardo D. (2014). Exosomal Lipids Induce Human Pancreatic Tumoral MiaPaCa-2 Cells Resistance through the CXCR4-SDF-1α Signaling Axis. Oncoscience.

[159] Shafi O., Rajpar R., Aakash, Waqas M., Haseeb M., Raveena, Kumari M., Kumar A., Kumari M., Yaqub M.D. (2024). NF-κB Mediated Disruption of Pancreatic Signaling Pathways and Its Implications for the Risk of Pancreatic Adenocarcinoma: A Systematic Review. medRxiv.

[160] Zhang H., Xing J., Dai Z., Wang D., Tang D. (2022). Exosomes: The Key of Sophisticated Cell-Cell Communication and Targeted Metastasis in Pancreatic Cancer. Cell Commun. Signal..

[161] Khasabova I.A., Khasabov S.G., Johns M., Juliette J., Zheng A., Morgan H., Flippen A., Allen K., Golovko M.Y., Golovko S.A. (2023). Exosome-Associated Lysophosphatidic Acid Signaling Contributes to Cancer Pain. Pain.

[162] Zhang Q., Deng T., Zhang H., Zuo D., Zhu Q., Bai M., Liu R., Ning T., Zhang L., Yu Z. (2022). Adipocyte-Derived Exosomal MTTP Suppresses Ferroptosis and Promotes Chemoresistance in Colorectal Cancer. Adv. Sci..

[163] Nishida-Aoki N., Izumi Y., Takeda H., Takahashi M., Ochiya T., Bamba T. (2020). Lipidomic Analysis of Cells and Extracellular Vesicles from High- and Low-Metastatic Triple-Negative Breast Cancer. Metabolites.

[164] Prendeville H., Lynch L. (2022). Diet, Lipids, and Antitumor Immunity. Cell. Mol. Immunol..

[165] Gupta P., Kadamberi I.P., Mittal S., Tsaih S., George J., Kumar S., Vijayan D.K., Geethadevi A., Parashar D., Topchyan P. (2022). Tumor Derived Extracellular Vesicles Drive T Cell Exhaustion in Tumor Microenvironment through Sphingosine Mediated Signaling and Impacting Immunotherapy Outcomes in Ovarian Cancer. Adv. Sci..

[166] Bai S., Wang Z., Wang M., Li J., Wei Y., Xu R., Du J. (2022). Tumor-Derived Exosomes Modulate Primary Site Tumor Metastasis. Front. Cell Dev. Biol..

[167] Olejarz W., Dominiak A., Żołnierzak A., Kubiak-Tomaszewska G., Lorenc T. (2020). Tumor-Derived Exosomes in Immunosuppression and Immunotherapy. J. Immunol. Res..

[168] Brzozowski J.S., Jankowski H., Bond D.R., McCague S.B., Munro B.R., Predebon M.J., Scarlett C.J., Skelding K.A., Weidenhofer J. (2018). Lipidomic Profiling of Extracellular Vesicles Derived from Prostate and Prostate Cancer Cell Lines. Lipids Health Dis..

[169] Cheng L., Zhang K., Qing Y., Li D., Cui M., Jin P., Xu T. (2020). Proteomic and Lipidomic Analysis of Exosomes Derived from Ovarian Cancer Cells and Ovarian Surface Epithelial Cells. J. Ovarian Res..

[170] Bestard-Escalas J., Reigada R., Reyes J., de la Torre P., Liebisch G., Barceló-Coblijn G. (2021). Fatty Acid Unsaturation Degree of Plasma Exosomes in Colorectal Cancer Patients: A Promising Biomarker. Int. J. Mol. Sci..

[171] Elmallah M.I.Y., Ortega-Deballon P., Hermite L., Pais-De-Barros J.-P., Gobbo J., Garrido C. (2022). Lipidomic Profiling of Exosomes from Colorectal Cancer Cells and Patients Reveals Potential Biomarkers. Mol. Oncol..

[172] Coviello G., Tutino V., Notarnicola M., Caruso M.G. (2014). Erythrocyte Membrane Fatty Acids Profile in Colorectal Cancer Patients: A Preliminary Study. Anticancer Res..

[173] Huang X., Yuan T., Tschannen M., Sun Z., Jacob H., Du M., Liang M., Dittmar R.L., Liu Y., Liang M. (2013). Characterization of Human Plasma-Derived Exosomal RNAs by Deep Sequencing. BMC Genom..

[174] Yuan T., Huang X., Woodcock M., Du M., Dittmar R., Wang Y., Tsai S., Kohli M., Boardman L., Patel T. (2016). Plasma Extracellular RNA Profiles in Healthy and Cancer Patients. Sci. Rep..

[175] Cheng L., Sharples R.A., Scicluna B.J., Hill A.F. (2014). Exosomes Provide a Protective and Enriched Source of miRNA for Biomarker Profiling Compared to Intracellular and Cell-Free Blood. J. Extracell. Vesicles.

[176] Xiao Y., Zhong J., Zhong B., Huang J., Jiang L., Jiang Y., Yuan J., Sun J., Dai L., Yang C. (2020). Exosomes as Potential Sources of Biomarkers in Colorectal Cancer. Cancer Lett..

[177] Liu X., Chen X., Zeng K., Xu M., He B., Pan Y., Sun H., Pan B., Xu X., Xu T. (2018). DNA-Methylation-Mediated Silencing of miR-486-5p Promotes Colorectal Cancer Proliferation and Migration through Activation of PLAGL2/IGF2/β-Catenin Signal Pathways. Cell Death Dis..

[178] Ji H., Chen M., Greening D.W., He W., Rai A., Zhang W., Simpson R.J. (2014). Deep Sequencing of RNA from Three Different Extracellular Vesicle (EV) Subtypes Released from the Human LIM1863 Colon Cancer Cell Line Uncovers Distinct Mirna-Enrichment Signatures. PLoS ONE.

[179] Matsumura T., Sugimachi K., Iinuma H., Takahashi Y., Kurashige J., Sawada G., Ueda M., Uchi R., Ueo H., Takano Y. (2015). Exosomal microRNA in Serum Is a Novel Biomarker of Recurrence in Human Colorectal Cancer. Br. J. Cancer.

[180] Ogata-Kawata H., Izumiya M., Kurioka D., Honma Y., Yamada Y., Furuta K., Gunji T., Ohta H., Okamoto H., Sonoda H. (2014). Circulating Exosomal microRNAs as Biomarkers of Colon Cancer. PLoS ONE.

[181] Liu Y.D., Zhuang X.P., Cai D.L., Cao C., Gu Q.S., Liu X.N., Zheng B.B., Guan B.J., Yu L., Li J.K. (2021). Let-7a Regulates EV Secretion and Mitochondrial Oxidative Phosphorylation by Targeting SNAP23 in Colorectal Cancer. J. Exp. Clin. Cancer Res..

[182] Qin R., Huang Y., Yao Y., Wang L., Zhang Z., Huang W., Su Y., Zhang Y., Guan A., Wang H. (2023). The Role and Molecular Mechanism of Metabolic Reprogramming of Colorectal Cancer by UBR5 through PYK2 Regulation of OXPHOS Expression Study. J. Biochem. Mol. Toxicol..

[183] Ren L., Meng L., Gao J., Lu M., Guo C., Li Y., Rong Z., Ye Y. (2023). PHB2 Promotes Colorectal Cancer Cell Proliferation and Tumorigenesis through NDUFS1-Mediated Oxidative Phosphorylation. Cell Death Dis..

[184] Qiu X., Wang A., Wang J., Zhang Z., Tao L. (2025). Mitochondrial Metabolic Reprogramming in Colorectal Cancer: Mechanisms of Resistance and Future Clinical Interventions. Cell Death Discov..

[185] Pang B., Wu H. (2025). Metabolic Reprogramming in Colorectal Cancer: A Review of Aerobic Glycolysis and Its Therapeutic Implications for Targeted Treatment Strategies. Cell Death Discov..

[186] Qin R., Fan X., Huang Y., Chen S., Ding R., Yao Y., Wu R., Duan Y., Li X., Khan H.U. (2024). Role of Glucose Metabolic Reprogramming in Colorectal Cancer Progression and Drug Resistance. Transl. Oncol..

[187] Fu F., Jiang W., Zhou L., Chen Z. (2018). Circulating Exosomal miR-17-5p and miR-92a-3p Predict Pathologic Stage and Grade of Colorectal Cancer. Transl. Oncol..

[188] Cooks T., Pateras I.S., Jenkins L.M., Patel K.M., Robles A.I., Morris J., Forshew T., Appella E., Gorgoulis V.G., Harris C.C. (2018). Mutant P53 Cancers Reprogram Macrophages to Tumor Supporting Macrophages via Exosomal miR-1246. Nat. Commun..

[189] Bao H., Peng Z., Cheng X., Jian C., Li X., Shi Y., Zhu W., Hu Y., Jiang M., Song J. (2023). GABA Induced by Sleep Deprivation Promotes the Proliferation and Migration of Colon Tumors through miR-223-3p Endogenous Pathway and Exosome Pathway. J. Exp. Clin. Cancer Res..

[190] Palmqvist R., Engarås B., Lindmark G., Hallmans G., Tavelin B., Nilsson O., Hammarström S., Hafström L. (2003). Prediagnostic Levels of Carcinoembryonic Antigen and CA 242 in Colorectal Cancer: A Matched Case-Control Study. Dis. Colon Rectum.

[191] Chen Y., Xie Y., Xu L., Zhan S., Xiao Y., Gao Y., Wu B., Ge W. (2017). Protein Content and Functional Characteristics of Serum-Purified Exosomes from Patients with Colorectal Cancer Revealed by Quantitative Proteomics. Int. J. Cancer.

[192] Campanella C., Rappa F., Sciumè C., Marino Gammazza A., Barone R., Bucchieri F., David S., Curcurù G., Caruso Bavisotto C., Pitruzzella A. (2015). Heat Shock Protein 60 Levels in Tissue and Circulating Exosomes in Human Large Bowel Cancer before and after Ablative Surgery. Cancer.

[193] Li J., Chen Y., Guo X., Zhou L., Jia Z., Peng Z., Tang Y., Liu W., Zhu B., Wang L. (2017). GPC1 Exosome and Its Regulatory miRNAs Are Specific Markers for the Detection and Target Therapy of Colorectal Cancer. J. Cell. Mol. Med..

[194] Liang Z., Liu H., Wang F., Xiong L., Zhou C., Hu T., He X., Wu X., Xie D., Wu X. (2019). LncRNA RPPH1 Promotes Colorectal Cancer Metastasis by Interacting with TUBB3 and by Promoting Exosomes-Mediated Macrophage M2 Polarization. Cell Death Dis..

[195] Sun B., Li Y., Zhou Y., Ng T.K., Zhao C., Gan Q., Gu X., Xiang J. (2019). Circulating Exosomal CPNE3 as a Diagnostic and Prognostic Biomarker for Colorectal Cancer. J. Cell. Physiol..

[196] Zhou G.-Y.-J., Zhao D.-Y., Yin T.-F., Wang Q.-Q., Zhou Y.-C., Yao S.-K. (2023). Proteomics-Based Identification of Proteins in Tumor-Derived Exosomes as Candidate Biomarkers for Colorectal Cancer. World J. Gastrointest. Oncol..

[197] Rai A., Greening D.W., Chen M., Xu R., Ji H., Simpson R.J. (2019). Exosomes Derived from Human Primary and Metastatic Colorectal Cancer Cells Contribute to Functional Heterogeneity of Activated Fibroblasts by Reprogramming Their Proteome. Proteomics.

[198] Sun B., Zhou Y., Fang Y., Li Z., Gu X., Xiang J. (2019). Colorectal Cancer Exosomes Induce Lymphatic Network Remodeling in Lymph Nodes. Int. J. Cancer.

[199] Liu J., Ren L., Li S., Li W., Zheng X., Yang Y., Fu W., Yi J., Wang J., Du G. (2021). The Biology, Function, and Applications of Exosomes in Cancer. Acta Pharm. Sin. B.

[200] Tauro B.J., Greening D.W., Mathias R.A., Mathivanan S., Ji H., Simpson R.J. (2013). Two Distinct Populations of Exosomes Are Released from LIM1863 Colon Carcinoma Cell-Derived Organoids. Mol. Cell. Proteom..

[201] Liu D., Sun J., Zhu J., Zhou H., Zhang X., Zhang Y. (2014). Expression and Clinical Significance of Colorectal Cancer Stem Cell Marker EpCAMhigh/CD44+ in Colorectal Cancer. Oncol. Lett..

[202] Welt S., Ritter G., Williams C., Cohen L.S., John M., Jungbluth A., Richards E.A., Old L.J., Kemeny N.E. (2003). Phase I Study of Anticolon Cancer Humanized Antibody A331. Clin. Cancer Res..

[203] Baptistella A.R., Salles Dias M.V., Aguiar S.J., Begnami M.D., Martins V.R. (2016). Heterogeneous Expression of A33 in Colorectal Cancer: Possible Explanation for A33 Antibody Treatment Failure. Anti-Cancer Drugs.

[204] Hafez F., Shakweer M., Sabry D., Tawfik A., El-Beah S., Elsheshtawy N., Shash L., Salama D., Gaballah A. (2024). TLR4, IgA and EpCAM Expression in Colorectal Cancer and Their Possible Association with Microbiota as a Pathogenic Factor; An Immunohistochemical and Genetic Study. Asian Pac. J. Cancer Prev..

[205] Mathivanan S., Lim J.W.E., Tauro B.J., Ji H., Moritz R.L., Simpson R.J. (2010). Proteomics Analysis of A33 Immunoaffinity-Purified Exosomes Released from the Human Colon Tumor Cell Line LIM1215 Reveals a Tissue-Specific Protein Signature. Mol. Cell. Proteom..

[206] Gastpar R., Gehrmann M., Bausero M.A., Asea A., Gross C., Schroeder J.A., Multhoff G. (2005). Heat Shock Protein 70 Surface-Positive Tumor Exosomes Stimulate Migratory and Cytolytic Activity of Natural Killer Cells. Cancer Res..

[207] Asea A., Kraeft S.K., Kurt-Jones E.A., Stevenson M.A., Chen L.B., Finberg R.W., Koo G.C., Calderwood S.K. (2000). HSP70 Stimulates Cytokine Production through a CD14-Dependant Pathway, Demonstrating Its Dual Role as a Chaperone and Cytokine. Nat. Med..

[208] Maqsood Q., Sumrin A., Saleem Y., Wajid A., Mahnoor M. (2024). Exosomes in Cancer: Diagnostic and Therapeutic Applications. Clin. Med. Insights Oncol..

[209] Huang T., Deng C.-X. (2019). Current Progresses of Exosomes as Cancer Diagnostic and Prognostic Biomarkers. Int. J. Biol. Sci..

[210] Ale Ebrahim S., Ashtari A., Zamani Pedram M., Ale Ebrahim N., Sanati-Nezhad A. (2020). Publication Trends in Exosomes Nanoparticles for Cancer Detection. Int. J. Nanomed..

[211] Shin H., Choi B.H., Shim O., Kim J., Park Y., Cho S.K., Kim H.K., Choi Y. (2023). Single Test-Based Diagnosis of Multiple Cancer Types Using Exosome-SERS-AI for Early Stage Cancers. Nat. Commun..

[212] Yang Z., Tian T., Kong J., Chen H. (2025). ChatExosome: An Artificial Intelligence (AI) Agent Based on Deep Learning of Exosomes Spectroscopy for Hepatocellular Carcinoma (HCC) Diagnosis. Anal. Chem..

[213] Lu D., Shangguan Z., Su Z., Lin C., Huang Z., Xie H. (2024). Artificial Intelligence-Based Plasma Exosome Label-Free SERS Profiling Strategy for Early Lung Cancer Detection. Anal. Bioanal. Chem..

[214] Tkach M., Théry C. (2016). Communication by Extracellular Vesicles: Where We Are and Where We Need to Go. Cell.

[215] Böing A.N., van der Pol E., Grootemaat A.E., Coumans F.A.W., Sturk A., Nieuwland R. (2014). Single-Step Isolation of Extracellular Vesicles by Size-Exclusion Chromatography. J. Extracell. Vesicles.

[216] Li P., Kaslan M., Lee S.H., Yao J., Gao Z. (2017). Progress in Exosome Isolation Techniques. Theranostics.

[217] Lener T., Gimona M., Aigner L., Börger V., Buzas E., Camussi G., Chaput N., Chatterjee D., Court F.A., del Portillo H.A. (2015). Applying Extracellular Vesicles Based Therapeutics in Clinical Trials—An ISEV Position Paper. J. Extracell. Vesicles.

[218] Willms E., Johansson H.J., Mäger I., Lee Y., Blomberg K.E.M., Sadik M., Alaarg A., Smith C.I.E., Lehtiö J., El Andaloussi S. (2016). Cells Release Subpopulations of Exosomes with Distinct Molecular and Biological Properties. Sci. Rep..

[219] He M., Crow J., Roth M., Zeng Y., Godwin A.K. (2014). Integrated Immunoisolation and Protein Analysis of Circulating Exosomes Using Microfluidic Technology. Lab. Chip.

[220] Ullah M., Qian N.P.M., Yannarelli G. (2021). Advances in Innovative Exosome-Technology for Real Time Monitoring of Viable Drugs in Clinical Translation, Prognosis and Treatment Response. Oncotarget.

[221] Mukerjee N., Bhattacharya A., Maitra S., Kaur M., Ganesan S., Mishra S., Ashraf A., Rizwan M., Kesari K.K., Tabish T.A. (2025). Exosome Isolation and Characterization for Advanced Diagnostic and Therapeutic Applications. Mater. Today Bio.

[222] Lee K.W.A., Chan L.K.W., Hung L.C., Phoebe L.K.W., Park Y., Yi K.-H. (2024). Clinical Applications of Exosomes: A Critical Review. Int. J. Mol. Sci..

[223] Youssef E., Palmer D., Fletcher B., Vaughn R. (2025). Exosomes in Precision Oncology and Beyond: From Bench to Bedside in Diagnostics and Therapeutics. Cancers.

[224] Welsh J.A., Goberdhan D.C.I., O’Driscoll L., Buzas E.I., Blenkiron C., Bussolati B., Cai H., Di Vizio D., Driedonks T.A.P., Erdbrügger U. (2024). Minimal Information for Studies of Extracellular Vesicles (MISEV2023): From Basic to Advanced Approaches. J. Extracell. Vesicles.

[225] Lipidomics Standards Initiative (LSI)—International Lipidomics Society. https://lipidomicssociety.org/interest_groups/lipidomics-standards-initiative-lsi/.

[226] Singh M., Tiwari P.K., Kashyap V., Kumar S. (2025). Proteomics of Extracellular Vesicles: Recent Updates, Challenges and Limitations. Proteomes.

[227] Ramirez M.I., Amorim M.G., Gadelha C., Milic I., Welsh J.A., Freitas V.M., Nawaz M., Akbar N., Couch Y., Makin L. (2018). Technical Challenges of Working with Extracellular Vesicles. Nanoscale.

[228] Xu C., Mannucci A., Esposito F., Oliveres H., Alonso-Orduña V., Yubero A., Fernández-Martos C., Salud A., Gallego J., Martín-Richard M. (2025). An Exosome-Based Liquid Biopsy Predicts Depth of Response and Survival Outcomes to Cetuximab and Panitumumab in Metastatic Colorectal Cancer: The EXONERATE Study. Clin. Cancer Res..

